# Circular RNA cVIM promotes hepatic stellate cell activation in liver fibrosis via miR-122-5p/miR-9-5p-mediated TGF-β signaling cascade

**DOI:** 10.1038/s42003-024-05797-3

**Published:** 2024-01-19

**Authors:** Zhenxu Zhou, Rongrong Zhang, Xinmiao Li, Weizhi Zhang, Yating Zhan, Zhichao Lang, Qiqi Tao, Jinglu Yu, Suhui Yu, Zhengping Yu, Jianjian Zheng

**Affiliations:** 1 Department of Hernia and Abdominal Wall Surgery, The First Affiliated Hospital of Wenzhou Medical University, Wenzhou, 325000 China; 2 Key Laboratory of Diagnosis and Treatment of Severe Hepato-Pancreatic Diseases of Zhejiang Province, The First Affiliated Hospital of Wenzhou Medical University, Wenzhou, 325000 China; 3 Department of Hepatobiliary Surgery, The First Affiliated Hospital of Wenzhou Medical University, Wenzhou, 325000 China; 4 Key Laboratory of Clinical Laboratory Diagnosis and Translational Research of Zhejiang Province, The First Affiliated Hospital of Wenzhou Medical University, Wenzhou, 325000 China

**Keywords:** Non-coding RNAs, miRNAs

## Abstract

Hepatic stellate cell (HSC) activation is considered as a central driver of liver fibrosis and effective suppression of HSC activation contributes to the treatment of liver fibrosis. Circular RNAs (circRNAs) have been reported to be important in tumor progression. However, the contributions of circRNAs in liver fibrosis remain largely unclear. The liver fibrosis-specific circRNA was explored by a circRNA microarray and cVIM (a circRNA derived from exons 4 to 8 of the vimentin gene mmu_circ_32994) was selected as the research object. Further studies revealed that cVIM, mainly expressed in the cytoplasm, may act as a sponge for miR-122-5p and miR-9-5p to enhance expression of type I TGF-β receptor (TGFBR1) and TGFBR2 and promotes activation of the TGF-β/Smad pathway, thereby accelerating the progression of liver fibrosis. Our results demonstrate a vital role for cVIM in promoting liver fibrosis progression and provide a fresh perspective on circRNAs in liver fibrosis.

## Abstract (web summary)

The circRNA cVIM may act as a sponge for miR-122-5p and miR-9-5p to enhance expression of TGFBR1 and TGFBR2 and promote activation of the TGF-β/Smad pathway, thereby accelerating the progression of liver fibrosis.

## Introduction

Liver fibrosis induced by long-standing liver injury is a dynamic process characterized by excessive accumulation of the extracellular matrix (ECM)^1^. Progressive liver fibrosis results in at least one million deaths annually via the development of cirrhosis, posing a considerable healthcare burden worldwide^2,3^. Furthermore, patients with liver cirrhosis are at significant risk of developing primary liver cancer, in particular, hepatocellular carcinoma^4–6^. However, no available anti-fibrogenic agents have been approved as therapy for fibrotic diseases to date.

Hepatic stellate cell (HSC) activation has been established as a central driver of liver fibrosis in experimental and human liver injury^7^. HSCs generally maintain a quiescent phenotype, developing into proliferative, migratory, and contractile myofibroblasts in fibrogenic liver^8,9^. Accumulating evidence has shown that activated HSCs are the main source of ECM production in liver fibrosis^10^. Regulation of the signaling molecules and pathways involved in HSC activation can effectively aid in controlling liver fibrosis progression^7^. For instance, following stimulation of peroxisome proliferator­activated receptor γ (PPARγ), activated HSCs revert to a quiescent phenotype, leading to the suppression of liver fibrosis^11^. Effective suppression of HSC activation, thus, presents a promising therapeutic strategy for liver fibrosis.

Circular RNAs (circRNAs), a novel class of RNAs generated from protein-coding genes via back splicing, are characterized by covalently closed loop structures without free 3′ or 5′ ends^12–14^. In this particular conformation, structural stability and resistance against exonucleolytic RNA decay are enhanced. Recent studies have shown that circRNAs, deregulated in various cancers, participate in tumor progression^15–17^. CircRNAs have been shown to play crucial roles in diverse biological processes, such as proliferation and differentiation^18,19^. However, the circRNAs involved in liver fibrosis and their specific functions remain to be established.

In the present study, we employed microarray analysis to examine circRNA expression patterns between carbon tetrachloride (CCl_4_)-treated and healthy control mice. Based on the initial analysis, a circRNA derived from exons 4 to 8 of the vimentin (VIM) gene (termed cVIM) was selected for subsequent experiments and its functions and mechanisms of action in liver fibrosis progression were extensively explored.

## Results

### Deregulation of circRNAs and upregulation of cVIM in liver fibrosis

**Fig. 1:**
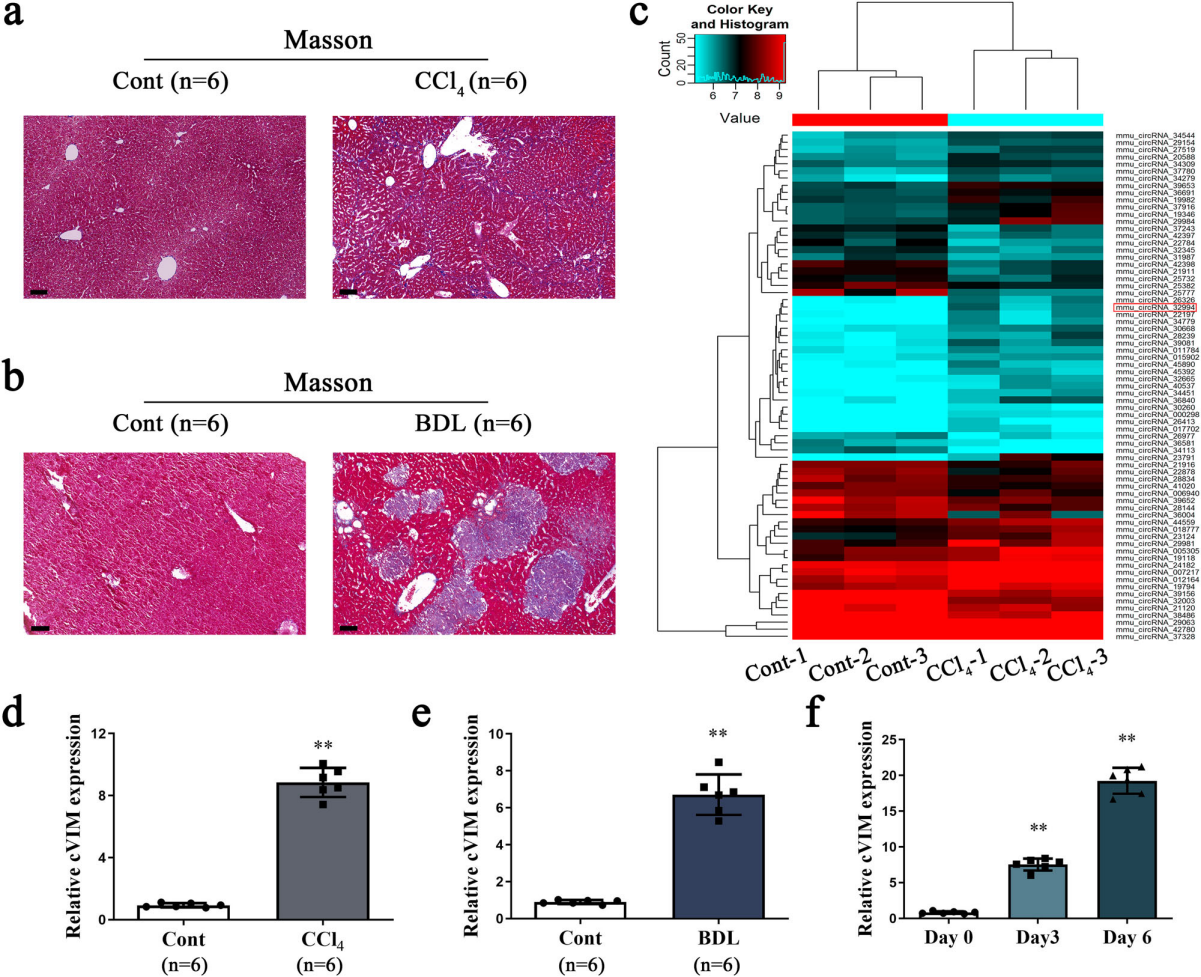
Deregulated circRNAs and up-regulation of cVIM in liver fibrosis. Masson staining was performed in CCl_4_ mice (**a**) and BDL mice (**b**), *n* = 6 mice per group. Scale bar, 100 μm. **c** Heat map for differentially expressed circRNAs analyzed by circRNA arraystar ChIP between the fibrotic tissues and the control tissues (*n* = 3 per group). Expression of cVIM in the fibrotic tissues in CCl_4_ (**d**) and BDL mice (**e**), *n* = 6 mice per group. **f** Expression of cVIM in primary HSCs isolated from healthy mice during culture days (*n* = 6 per group). Each value is the mean ± SD of six independent experiments. **P < 0.01 compared to the control.

To identify the potential liver fibrosis-related circRNAs, two experimental mouse models of hepatic fibrosis [CCl_4_ and Bile duct ligation (BDL)] were generated. Masson staining revealed enhanced collagen expression in CCl_4_- and BDL-treated mice, compared with their control counterparts, indicating successful establishment of animal liver fibrosis models (Fig. 1a, b). To investigate the differences in circRNA expression profiles between fibrotic and normal livers, circRNA microarray for total RNA extracts isolated from mouse livers treated with CCl_4_ or oil (*n* = 3 per group) was performed. Differences were considered significant when changes in circRNA levels were >1.5 fold between the experimental and control groups (*P* < 0.05). Overall, 44 circRNAs were upregulated and 27 downregulated in fibrotic liver (Fig. 1c and Supplementary Table 3). Among the upregulated circRNAs, only three (mmu_circRNA_39081, mmu_circRNA_34779, and mmu_circRNA_32994) showed >2.0 fold change between the experimental and control groups (*P* < 0.025), which was further confirmed in primary HSCs isolated from CCl_4_-treated mice. In particular, expression of mmu_circRNA_32994 was markedly enhanced in isolated cells (Supplementary Fig. 1A). Accordingly, we selected the circRNA derived from the *Vim* gene (ID: mmu_circRNA_32994, termed cVIM) for further experiments. Our results confirmed a significant increase in cVIM in both the CCl_4_ and BDL groups (Fig. 1d, e). Expression of cVIM was additionally examined in primary HSCs isolated from healthy controls. As expected, enhanced cVIM was observed in HSCs during culture times (Fig. 1f). The cVIM level was higher in primary HSCs than primary hepatocytes (Supplementary Fig. 1B). In addition, primary HSCs isolated from CCl_4_-treated mice at different time-points showed increased levels of cVIM (Supplementary Fig. 1C). The similar results could be also found in isolated primary hepatocytes (Supplementary Fig. 1D). The finding that cVIM, upregulated in activated HSCs, indicates a potential role in liver fibrosis.

### Characterization of cVIM

**Fig. 2:**
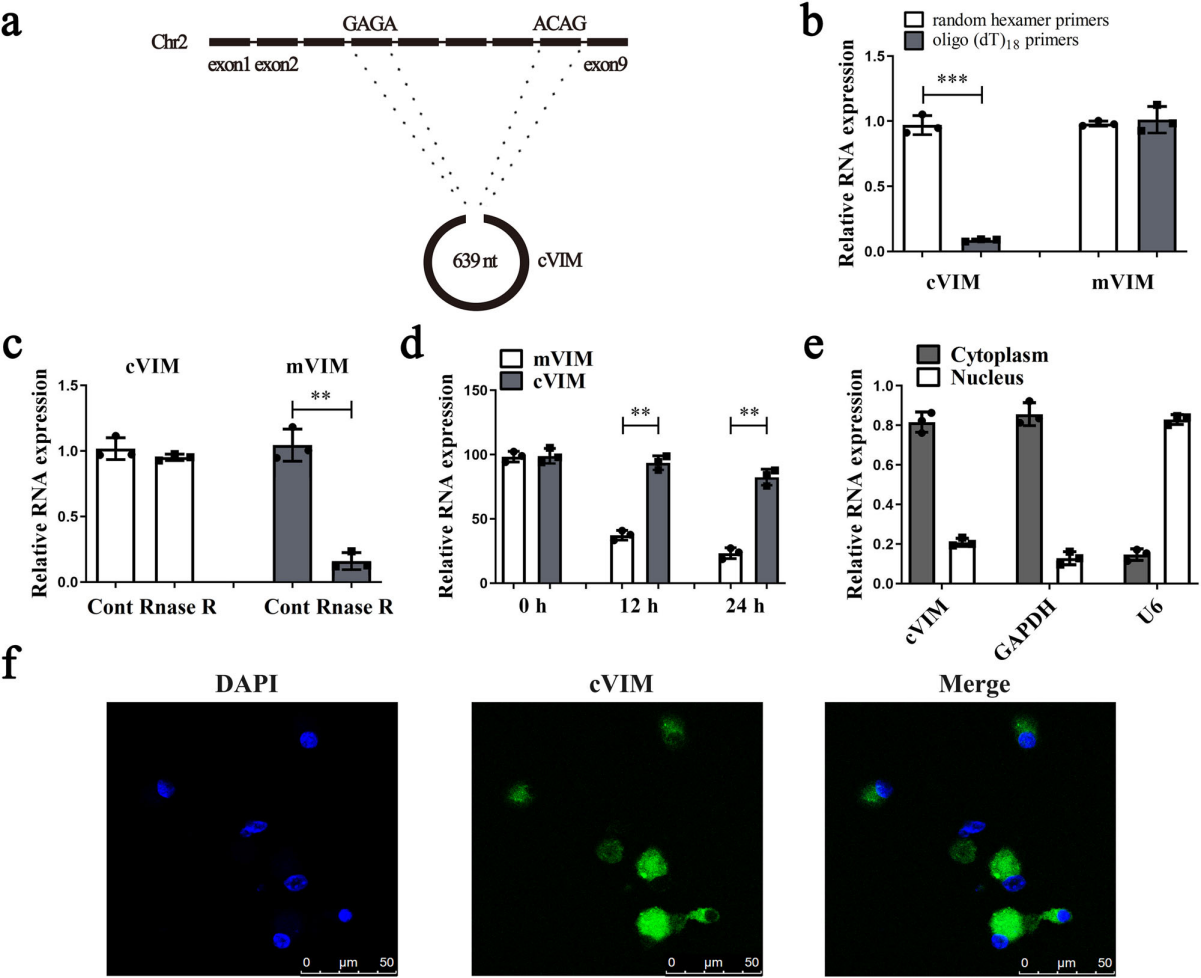
The characteristics of cVIM. Primary 1-day-old HSCs were isolated from CCl_4_-treated mice. **a** Scheme illustrating the production of cVIM. **b** Random hexamer or oligo (dT)_18_ primers were applied to perform the reverse transcription experiments and qRT-PCR was used to detect cVIM expression in primary 1-day-old HSCs (*n* = 3 per group). The random hexamer primers group was used as the control. **c** Relative cVIM expression was detected in primary 1-day-old HSCs after Rnase R treatment (*n* = 3 per group). **d** Relative cVIM expression was detected in primary 1-day-old HSCs by qRT-PCR after Actinomycin D at the indicated time points (*n* = 3 per group). **e** cVIM was mainly expressed in the cytoplasm in primary 1-day-old HSCs (*n* = 3 per group). GAPDH and U6 were applied as positive controls in the cytoplasm and nucleus, respectively. **f** RNA FISH for cVIM in primary 1-day-old HSCs (*n* = 3 per group). DAPI stained nuclei blue. Scale bar, 50 μm. Each value is the mean ± SD of three independent experiments. **P < 0.01 and ***P < 0.001 compared to the control.

cVIM is generated from exons 4 and 8 of the *Vim* gene via back-splicing (639 nt) (Fig. 2a). Sanger sequencing was initially performed to confirm the back-splice junction sequences (Supplementary Fig. 2), and subsequently, circular characteristics of cVIM were validated. RNA from primary HSCs was applied for reverse transcription experiments using either random hexamer or oligo (dT)_18_ primers. Compared with the random hexamer group, expression of cVIM was markedly reduced in the oligo (dT)_18_ group (Fig. 2b). In contrast, no significant changes were observed in mRNA of Vimentin (mVIM) between random hexamer and oligo (dT)_18_ groups. RNase R, a highly processive 3′ to 5′ exoribonuclease, digests linear RNAs. As shown in Fig. 2c, RNase R treatment led to a reduction in mVIM, but had no effect on cVIM levels. Measurement of the half-lives of cVIM and mVIM in cells after actinomycin D treatment disclosed higher stability of cVIM (Fig. 2d). Quantitative real-time PCR (qRT-PCR) findings showed predominant distribution of cVIM in the cytoplasm (Fig. 2e). As expected, RNA Fluorescence in situ hybridization (FISH) data on cVIM were consistent with the above findings (Fig. 2f). The collective results clearly demonstrate that cVIM is a circular RNA upregulated in liver fibrosis.

### Loss of cVIM inhibits progression of liver fibrosis in vitro and in vivo

**Fig. 3:**
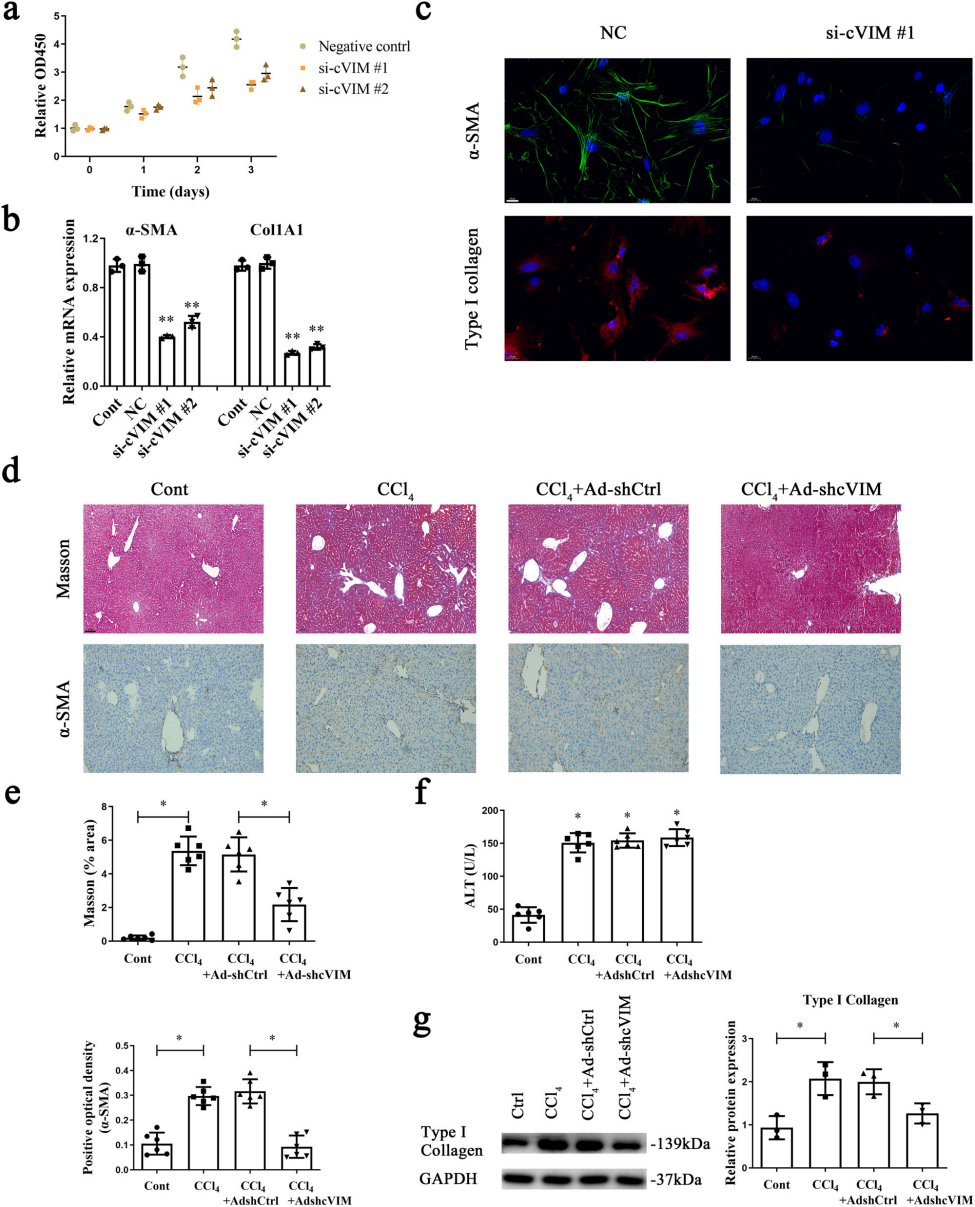
Loss of cVIM inhibits the progression of liver fibrosis in vitro and in vivo. Primary 1-day-old HSCs isolated from CCl_4_-treated mice were transfected with cVIM siRNA using Lipofectamine RNAiMAX for 24 h, 48 h and 72 h. **a** CCK8 assay showed the inhibitory role of loss of cVIM in HSCs (*n* = 3 per group). **b** mRNA expressions of α-SMA and Col1A1 (*n* = 3 per group). **c** Immunofluorescence staining for α-SMA (green) and type I collagen (red) were evaluated by confocal laser microscopy (*n* = 3 per group). DAPI stained the nuclei blue. The scale bar represents 20 μm. **d** Masson staining and α-SMA immunohistochemistry in CCl_4_ mice after cVIM knockdown (*n* = 6 per group). The scale bar represents 100 μm. **e** Analysis of Masson staining and α-SMA immunohistochemistry (*n* = 6 per group). **f** ALT value (*n* = 6 per group). **g** Type I collagen expression in vivo (*n* = 3 per group). Each value is the mean ± SD of three independent experiments. *P < 0.05 and **P < 0.01 compared to the control.

To establish whether cVIM plays a role in liver fibrosis progression, cVIM silencing was performed in vitro and in vivo. As illustrated in Supplementary Fig. 3A, the cVIM level was significantly reduced following treatment with specific siRNA. In CCl_4_ mice subjected to shRNA against cVIM (Ad-shcVIM) treatment, we observed a marked reduction in cVIM (Supplementary Fig. 3B). In primary HSCs isolated from CCl_4_ mice subjected to Ad-shcVIM treatment, reduction in cVIM was evident (Supplementary Fig. 3C), indicating silencing in vivo. The similar results were observed in primary hepatocytes isolated from CCl_4_ mice after Ad-shcVIM treatment (Supplementary Fig. 3D). Moreover, silencing of cVIM had no effect on the level of Vimentin mRNA in vitro and in vivo (Supplementary Fig. 3E). Downregulation of cVIM clearly induced a decrease in HSC proliferation (Fig. 3a). Further examination of the effects of cVIM on HSC transdifferentiation and collagen expression revealed that cVIM caused a reduction in not only α-smooth muscle actin (α-SMA), but also alpha-1(I) collagen (Col1A1) expression (Fig. 3b). Immunofluorescence analysis confirmed the suppressive effects of loss of cVIM on α-SMA and type I collagen (Fig. 3c). In mice, CCl_4_ treatment triggered an increase in type I collagen level, which was inhibited upon loss of cVIM (Fig. 3d, e). Immunohistochemical imaging of α-SMA showed similar results (Fig. 3d, e), indicating an inhibitory role of loss of cVIM in vivo during liver fibrosis. Loss of cVIM had no effect on ALT value caused by CCl_4_ (Fig. 3f). As expected, western blot experiments confirmed that increased type I collagen by CCl_4_ was suppressed by loss of cVIM (Fig. 3g). Additionally, cVIM knockdown induced an increase in MMP2 and a reduction in Col3A1 and TIMP1 in CCl_4_-treated mice (Supplementary Fig. 3F). Likewise, pro-fibrogenic cytokines such as IL-6 and TGF-β1 were obviously reduced by cVIM knockdown in vitro and in vivo (Supplementary Fig. 3G, H). Taken together, our results suggest that downregulation of cVIM contributes to suppression of liver fibrosis.

### cVIM acts as a sponge for miR-122-5p and miR-9-5p

**Fig. 4:**
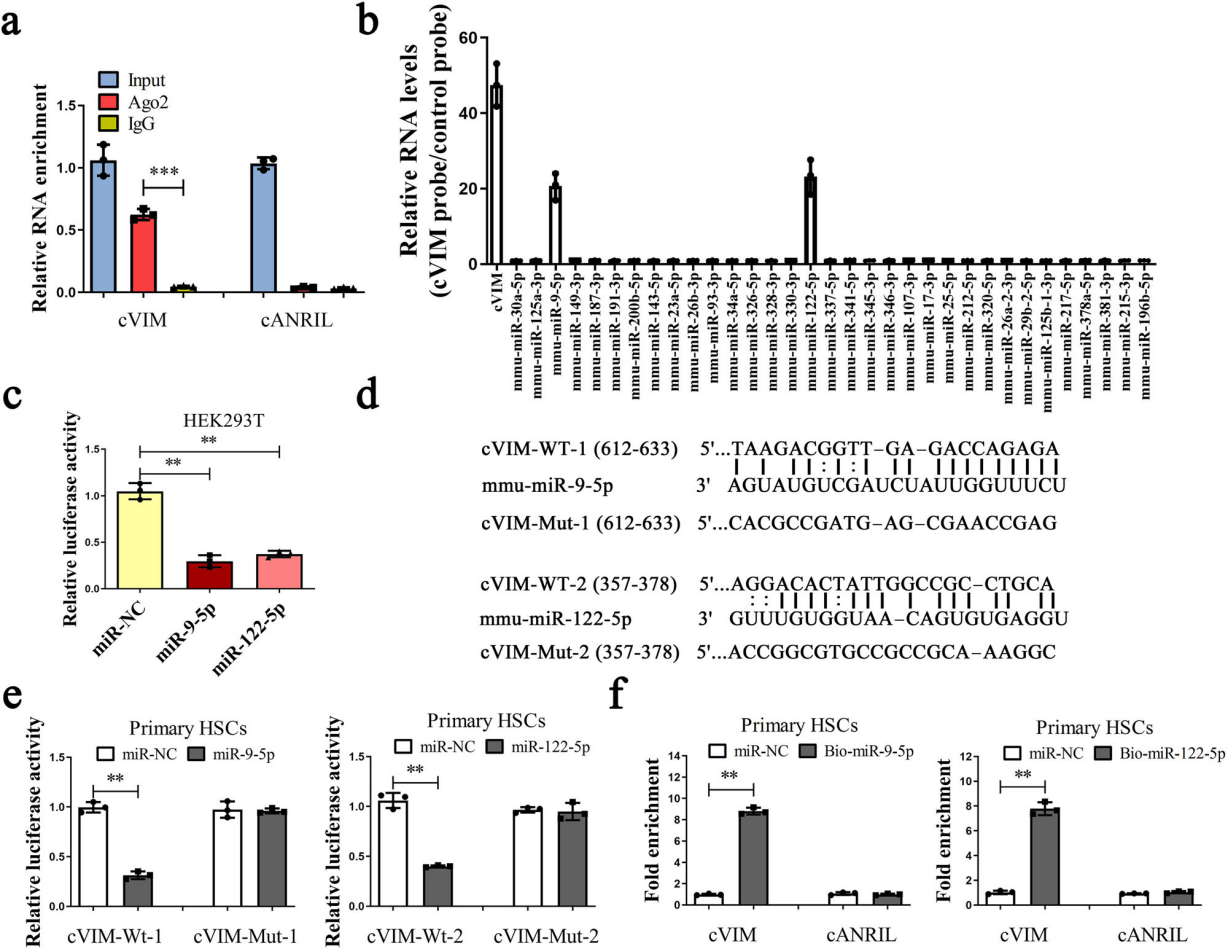
cVIM may act as a sponge for miR-122-5p and miR-9-5p. **a** RIP experiments (*n* = 3 per group). **b** circRIP was performed in cVIM-overexpressing HSCs using a cVIM-specific probe and control probe, respectively (*n* = 3 per group). The enrichments of cVIM and miRNAs were examined by qRT-PCR and normalized to the control probe. **c** The luciferase activity of luc-cVIM in HEK-293T cells with miR-122-5p or miR-9-5p mimics (*n* = 3 per group). **d** Scheme illustrating the putative binding sites of miR-122-5p and miR-9-5p with respect to cVIM. **e** The luciferase activity of luc-cVIM or luc-cVIM-mutant in HEK-293T cells after co-transfection with miR-122-5p or miR-9-5p (*n* = 3 per group). **f** Pull-down assay to validate the direct interaction between cVIM and miR-122-5p/miR-9-5p (*n* = 3 per group). Bio-miR-NC is not complementary to cVIM. Each value is the mean ± SD of three independent experiments. **P < 0.01 and ***P < 0.001.

Accumulating evidence suggests that circRNAs contribute to progression of human diseases through miRNA sponge effects. Due to the cytoplasmic location of cVIM, we further determined whether it acts as a binding platform for miRNAs. To this end, a RNA binding protein immunoprecipitation (RIP) experiment with an antibody against Argonaute-2 (Ago2) was performed. Notably, cVIM expression was enhanced by the Ago2 antibody, but not cANRIL (a circRNA reported not to bind to Ago2) (Fig. 4a). Next, bioinformatic analysis was performed to identify potential miRNAs interacting with cVIM. Application of miRanda, a miRNA target prediction tool, led to the identification of 58 potential binding miRNAs. Among these, interactions with cVIM were predicted for 33 miRNAs using RNAhybrid software (Supplementary Table 4). With the aid of probes against cVIM, circRNA in vivo precipitation (circRIP) was performed to obtain cVIM-associated RNAs and expression of the 33 candidate miRNAs was examined. Our results showed obvious enrichment of cVIM, miR-9-5p, and miR-122-5p, but not the other miRNAs (Fig. 4b), suggesting that miR-9-5p and miR-122-5p interact with cVIM and play a role in liver fibrosis. To confirm interactions between cVIM and miR-9-5p/miR-122-5p, the luciferase activity assay was performed. Both miR-9-5p and miR-122-5p clearly induced a reduction in luciferase activity (Fig. 4c). Subsequent mutation of the target sites for miR-9-5p/miR-122-5p (Fig. 4d) and transfection in the cVIM-Mut luciferase reporter led to no significant changes (Fig. 4e). To further confirm the interactions between cVIM and miR-9-5p/miR-122-5p, pull-down assays were performed. Biotinylated miR-122-5p/miR-9-5p (Bio-miR-122-5p/ Bio-miR-9-5p) led to significant enrichment of cVIM, but not cANRIL (Fig. 4f). Double FISH analysis confirmed co-localization of cVIM and miR-9-5p/miR-122-5p in the cytoplasm (Supplementary Fig. 4A). Next, we examined whether cVIM and miR-9-5p/miR-122-5p could be digested by each other. Expression of cVIM was not affected by a miR-9-5p/miR-122-5p mimic or inhibitor (Supplementary Fig. 4B). In keeping with this finding, overexpression or silencing of cVIM resulted in no significant changes in miR-9-5p and miR-122-5p (Supplementary Fig. 4B). These data suggest that cVIM could act as a sponge for miR-122-5p and miR-9-5p.

### cVIM promotes HSC activation via miR-122-5p/miR-9-5p-mediated TGF-β pathway

**Fig. 5:**
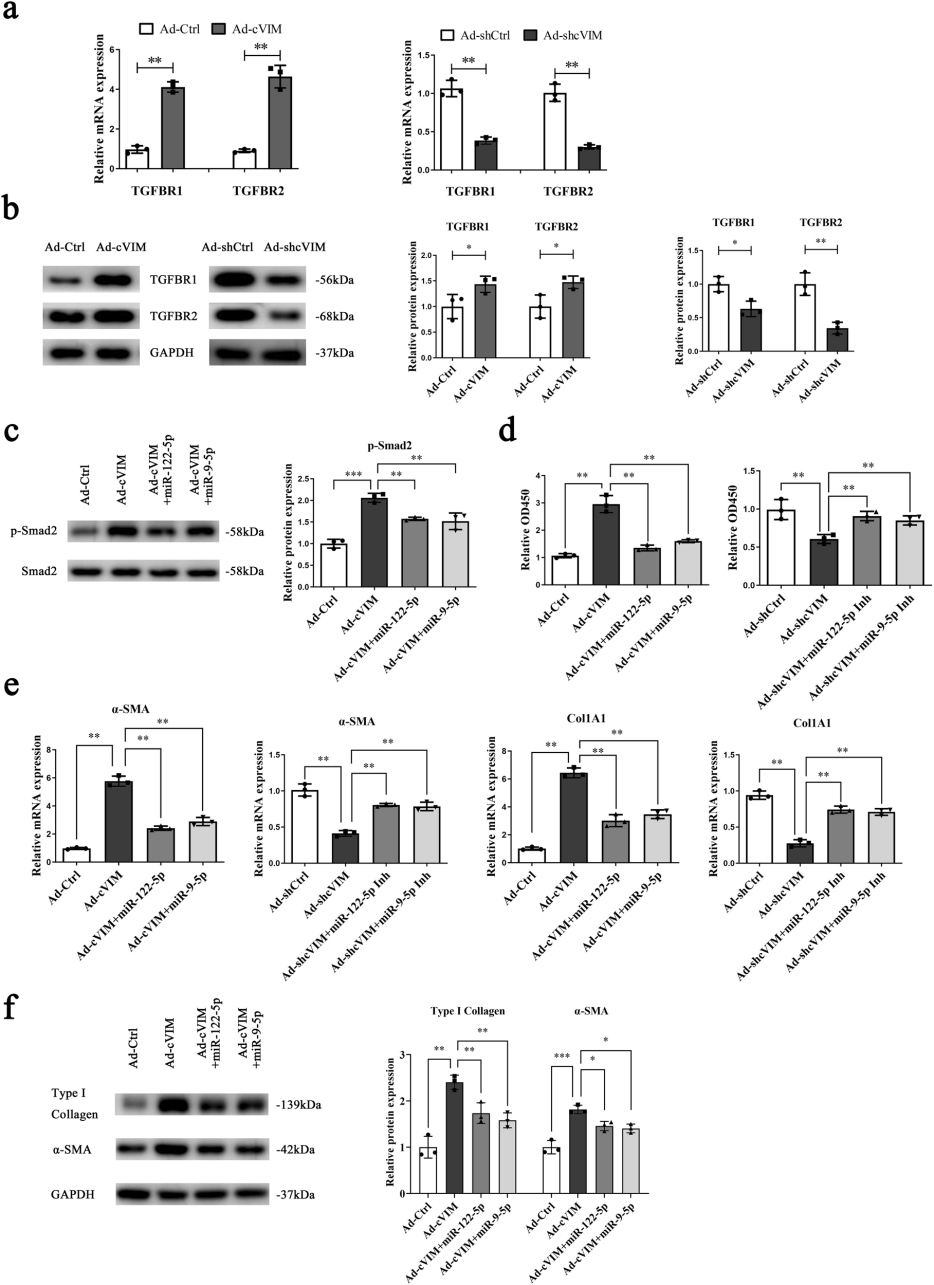
cVIM promotes HSC activation via miR-122-5p/miR-9-5p-midaited TGF-β pathway. Primary 1-day-old HSCs isolated from CCl_4_-treated mice were transduced with Ad-cVIM for 48 h and then transfected with miR-122-5p/miR-9-5p mimics for additional 24 h. In addition, cells were transduced with Ad-shcVIM for 48 h and then transfected with miR-122-5p/miR-9-5p inhibitor for additional 24 h. qRT-PCR (**a**) and Western blotting (**b**) analysis showed the mRNA and protein expressions of TGFBR1 and TGFBR2 after overexpressing or silencing cVIM (*n* = 3 per group). **c** p-Smad2 level in cVIM overexpressing-HSCs after co-transfection with miR-122-5p or miR-9-5p mimics (*n* = 3 per group). **d** Cell proliferation in cVIM overexpressing-HSCs after co-transfection with miR-122-5p or miR-9-5p mimics and cells with loss of cVIM after co-transfection with miR-122-5p or miR-9-5p inhibitor (*n* = 3 per group). **e** Col1A1 and α-SMA mRNA in cVIM overexpressing-HSCs after co-transfection with miR-122-5p or miR-9-5p mimics and cells with loss of cVIM after co-transfection with miR-122-5p or miR-9-5p inhibitor (*n* = 3 per group). **f** Type I collagen in cVIM overexpressing-HSCs after co-transfection with miR-122-5p or miR-9-5p mimics (*n* = 3 per group). Each value is the mean ± SD of three independent experiments. *P < 0.05, **P < 0.01 and ***P < 0.001.

To investigate the molecular mechanism of action of cVIM in liver fibrosis, a pathway reporter array was performed to identify the cVIM-related pathways. As shown in Supplementary Fig. 5A, TGF-β signaling was obviously activated by cVIM. Both miR-9-5p and miR-122-5p are reported to play anti-fibrotic roles in liver via targeting the TGF-β pathway^20–22^. Several members of the TGF-β pathway have been identified as targets of miR-9-5p and miR-122-5p (Supplementary Table 5). We hypothesized that cVIM promotes activation of HSCs by protecting these targets against downregulation by miR-122-5p and miR-9-5p. To examine this theory, we overexpressed miR-122-5p and miR-9-5p, and assessed the expression of their respective targets (Supplementary Table 5). As expected, type I TGF-β receptor (TGFBR1), TGFBR2 and NADPH oxidase 4 (NOX4) were downregulated upon overexpression of miR-9-5p. Moreover, Kruppel-like factor 6 (KLF6), TGF-β1, TGFBR2, serum response factor (SRF) and fibronectin 1 (FN1) were downregulated upon overexpression of miR-122-5p (Supplementary Fig. 5B). Next, the expression patterns of these target molecules were examined after overexpression or silencing of cVIM. Notably, TGFBR1 and TGFBR2 showed the most significant upregulation and downregulation during cVIM overexpression and depletion, respectively (Supplementary Fig. 5C). TGFBR1 and TGFBR2 are targets of miR-9-5p. Moreover, KLF6 is a transcriptional activator of TGFBR1 and TGFBR2 in liver fibrosis^23,24^, leading to indirect targeting by miR-122-5p. Based on the above findings, we focused on TGFBR1 and TGFBR2, the common targets of miR-9-5p and miR-122-5p. Overexpression of cVIM induced a significant increase in TGFBR1 and TGFBR2 mRNA and, conversely, its silencing suppressed TGFBR1 and TGFBR2 mRNA (Fig. 5a). Consistent with data on mRNA expression, TGFBR1 and TGFBR2 protein levels were enhanced by cVIM overexpression and inhibited by cVIM knockdown (Fig. 5b). Notably, exogenous miR-9-5p or miR-122-5p suppressed expression of both TGFBR1 and TGFBR2, which was restored by cVIM (Supplementary Fig. 6A, B). Activation of the TGF-β pathway in cVIM-expressing cells following miR-9-5p or miR-122-5p transfection was further examined. As expected, p-Smad2 induced by cVIM was attenuated by miR-9-5p or miR-122-5p overexpression (Fig. 5c). Conversely, loss of cVIM suppressed p-Smad2, which was blocked by both miR-9-5p inhibitor and miR-122-5p inhibitor (Supplementary Fig. 6C). Functionally, cell proliferation experiments showed that overexpression of cVIM promoted HSC growth, which was markedly inhibited by miR-9-5p or miR-122-5p, and vice versa (Fig. 5d). qRT-PCR and western blot analyses disclosed that overexpression of cVIM enhanced α-SMA and Col1A1 levels, which was effectively blocked by miR-9-5p or miR-122-5p (Fig. 5e, f) and vice versa (Fig. 5e and Supplementary S6C). These collective observations suggest that cVIM accelerates HSC activation, at least partly, through the miR-9-5p/miR-122-5p-mediated TGF-β pathway.

### cVIM transcription is activated by Sp1

**Fig. 6:**
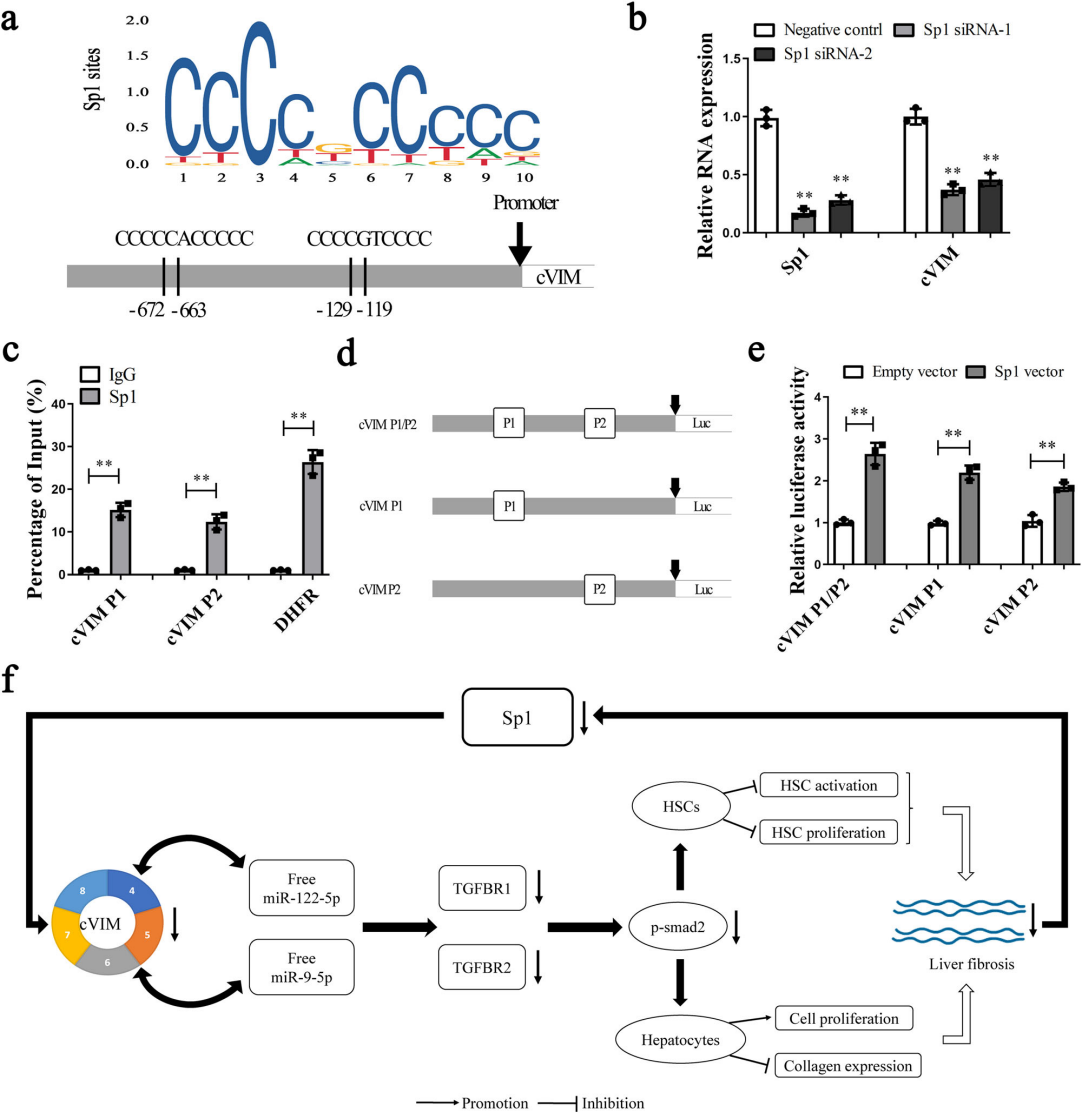
Sp1 activates cVIM expression in liver fibrosis. Primary 1-day-old HSCs isolated from CCl_4_-treated mice were transfected with Sp1 siRNA using Lipofectamine RNAiMAX for 48 h. **a** Sp1 binding site prediction in the cVIM promoter region using JASPAR. **b** qRT-PCR analysis of cVIM and Sp1 expression in HSCs after Sp1 siRNA or negative control treatment (*n* = 3 per group). **c** ChIP-qPCR analysis of Sp1 occupancy in the cVIM promoter in HSCs (*n* = 3 per group). DHFR was used as positive control and IgG was used as a negative control. **d** Construction of the luciferase reporter vector cVIM-P1/P2 (containg all Sp1 binding sites), cVIM-P1 (containg −672 and −663 binding sites) and cVIM-P2 (containg −129 and −119 binding sites). **e** Luciferase assays of the cells indicated that were transfected with cVIM-P1/P2, cVIM-P1, cVIM-P2 vectors, the Sp1 vector, or an empty vector (*n* = 3 per group). **f** Summary of the regulation and mechanism of cVIM in liver fibrosis. Each value is the mean ± SD of three independent experiments. **P < 0.01 compared to the control.

Next, the regulatory mechanism of cVIM transcription was investigated. Using the JASPAR and AnimalTFDB online databases, the transcription factor, Sp1, predicted to interact with the cVIM promoter with high scores, was selected for analysis. As shown in Fig. 6a, two predicted binding sites of Sp1 were identified in the cVIM promoter. In HSCs transfected with Sp1 siRNA, the cVIM level was clearly reduced (Fig. 6b). Two primers encompassing the Sp1 binding sites were designed and Chromatin immunoprecipitation (ChIP) assays performed to validate these sites. Our data confirmed interactions of Sp1 with these sites (Fig. 6c). As shown in Fig. 6d, luciferase reporter plasmids, including cVIM-P1/P2 (containing all binding sites), cVIM-P1 (positions −672 and −663) and cVIM-P2 (positions −129 and −119) were constructed. While Sp1 bound both P1 and P2 sites, higher luciferase activity was evident with the luciferase reporter containing all binding sites, compared with those containing only P1 or P2 (Fig. 6e). We additionally investigated whether cVIM affects Sp1 expression. Overexpression of cVIM promoted Sp1 level while its loss led to suppression of Sp1 (Supplementary Fig. 7A, B). Upregulation of Sp1 was found in activated HSCs of the CCl_4_-treated mouse model as well as CHB patients with liver fibrosis, suggesting a positive correlation between Sp1 expression and cVIM level (Supplementary Fig. 7C, D). Our data suggest that cVIM transcription is activated by Sp1, with a positive feedback loop between cVIM and Sp1.

### Effects of loss of cVIM on hepatocytes

Due to the reason that cVIM was significantly reduced in CCl_4_ mice after Ad-shcVIM treatment (Supplementary Fig. 3D), the effects of loss of cVIM were examined in primary hepatocytes. It was found that loss of cVIM led to a significant reduction in the mRNA expressions of TGFBR1 and TGFBR2 (Supplementary Fig. 8A). In line with it, the results of western blotting confirmed a reduction in the protein expressions of TGFBR1 and TGFBR2 in cells after Ad-shcVIM treatment (Supplementary Fig. 8B). Interestingly, silencing of cVIM led to an increase in cell proliferation in hepatocytes, which was inhibited by miR-122-5p or miR-9-5p inhibitor (Supplementary Fig. 8C). Accordingly, reduced collagen caused by loss of cVIM was blocked down by miR-122-5p or miR-9-5p inhibitor (Supplementary Fig. 8D, E). Notably, reduced p-Smad2 induced by loss of cVIM was suppressed by both miR-9-5p and miR-122-5p inhibitor (Supplementary Fig. 8E). Combined with these, our results suggest that cVIM-miR-122-5p/miR-9-5p-TGF-β signaling cascade participates in the progression of both HSCs and hepatocytes. As shown in Supplementary Fig. 8F, Sp1 down-regulation induced a reduction in cVIM expression, indicating that Sp1-mediated cVIM expression was also involved in hepatocytes.

### cVIM is a potential biomarker of liver fibrosis

In a circular RNA interactome (a database for circRNA, https://circinteractome.nia.nih.gov/Circular_RNA/circular_rna.html), 25 circRNA splices derived from the human vimentin (VIM) gene were validated using the circBase database. These circRNA levels were measured in the liver tissues of a cohort of 15 chronic hepatitis B (CHB) patients and 15 healthy controls. Our results showed higher levels of hsa_circ_0017884 than other circRNAs in CHB patients, compared with healthy controls (Supplementary Fig. 9A), with similar findings in CHC patients (Supplementary Fig. 9B). Accordingly, hsa-cVIM (hsa_circ_0017884) was selected for subsequent experiments and its expression measured in sera in another cohort of 110 CHB patients and 110 healthy controls. Consistently, hsa-cVIM was significantly upregulated in CHB patients (Supplementary Fig. 9C and Supplementary Table 1). To ascertain whether the serum hsa-cVIM level could serve as a potential diagnostic marker for liver fibrosis in CHB patents, receiver operating characteristic (ROC) curve analysis was performed. Based on serum hsa-cVIM levels, CHB patients with liver fibrosis were effectively differentiated from healthy controls, with area under the ROC curve (AUC) of ROC curve of 0.895 [95% confidence interval (CI): 0.847 to 0.933] (Supplementary Fig. 9D). At a cut-off value of 1.10, sensitivity and specificity were 100.0% and 72.7%, respectively. In addition, to explore the association between serum hsa-cVIM and fibrosis stage, CHB patents were divided into three groups according to fibrosis score, specifically, low (0–1), medium (2–4) and high (5–6). We observed a significant increase in hsa-cVIM with increasing fibrosis scores, indicative of a positive correlation (Supplementary Fig. 9E). Notably, there was a negative correlation between cVIM and miR-9-5p as well as miR-122-5p in liver samples from CHB patients (Supplementary Fig. 9F). Likewise, we also observed a positive correlation between cVIM and TGFBR1 as well as TGFBR2 (Supplementary Fig. 9F).

## Discussion

In this study, we screened differentially expressed circRNAs between fibrotic and control tissues via circRNA microarray and subsequently identified cVIM as a potential mediator in the development of liver fibrosis. Our data showed upregulation of cVIM in liver fibrosis and contributory effects on HSC activation. Additionally, the upregulation of cVIM was due, at least in part, to activation of Sp1 in liver fibrosis.

Although several circRNAs have been identified in the literature, only a few with biological relevance have been characterized to date. Accumulating evidence has demonstrated key roles of circRNAs in many human diseases^25,26^. CircRNAs are involved in regulation of gene expression via activity as competing endogenous RNAs (ceRNAs)^27^. Experiments in this study showed that cVIM is mainly expressed in the cytoplasm. It is known that miR-122-5p and miR-9-5p may play an inhibitory role in liver fibrosis. It was found that miR-122-5p and miR-9-5p were decreased in the fibrotic livers (Supplementary Fig. 4C), which may be associated with epigenetical regulation^20,28^. cVIM contains conserved miR-122-5p as well as miR-9-5p target sites, as validated using pulldown, RIP, luciferase and FISH analyses. Furthermore, expression of TGFBR1 and TGFBR2 (common targets of miR-122-5p and miR-9-5p) was positively regulated by cVIM. Our results collectively suggest that cVIM acts as a sponge for miR-122-5p and miR-9-5p to enhance expression of TGFBR1 and TGFBR2, leading to the phosphorylation of Smad2 (a downstream mediator of TGF-β signaling), which finally promotes TGF-β/Smad pathway and the progression of liver fibrosis (Fig. 6f). To date, the potential involvement of cVIM in human disease has rarely been explored. A recent investigation showed that cVIM serves as a valuable predictor for acute myeloid leukemia development and prognosis^29^. To our knowledge, the current study is the first to demonstrate a pro-fibrotic role of cVIM in liver fibrosis.

Biogenesis of circRNAs requires spliceosomal machinery and is modulated by both *cis* complementary sequences and trans-acting factors^30^. For example, the immune factors NF90 and/or NF110 promote circRNA formation in general by directly binding to IRAlus formed in nascent pre-mRNA^31^. In this investigation, online transcription factor binding site prediction analysis showed the existence of Sp1 binding sites in the cVIM promoter region. Sp1, an important transcription factor in liver fibrosis, is required for the regulation of fibrosis-related genes, such as CTGF, Col1A1, laminin and Smads^32–36^. Data from luciferase reporter and ChIP assays further provided evidence that Sp1 binds directly to the cVIM promoter region and activates its transcription. In addition, both Sp1 and cVIM were increased in vivo during liver fibrosis, indicating a positive correlation between Sp1 expression and cVIM level. Our collective results suggest that cVIM overexpression in liver fibrosis is partly attributable to binding of the transcription factor, Sp1, to its promoter region and stimulating transcription. Recent studies has demonstrated that up-regulation of TGF-β pathway can enhance the expression of Sp1^37^. Herein, cVIM enhances the TGF-β pathway. Therefore, cVIM may also upregulate the expression of Sp1 via TGF-β pathway.

Vimentin, a major structural component of intermediate filaments, participates in numerous vital biological functions, such as cell migration and proliferation^38^. Vimentin has been identified as a mesenchymal cell marker during epithelial mesenchymal transition (EMT). Activation of HSCs in liver fibrosis is considered as an EMT process^39,40^. Transition from quiescent HSCs during culture into myofibroblastic HSC is associated with reduced E-cadherin and a concomitant increase in vimentin. Upregulation of vimentin is reported to contribute to liver fibrosis progression^41^. In our experiments, both cVIM and mVIM could be upregulated after overexpression of Sp1 (Supplementary Fig. 10). Sp1 regulates cVIM expression via its site P1 and P2. Sp1 may also regulate VIM expression via this manner and further studies may be performed in future. Interestingly, cVIM overexpression led to activation TGF-β signaling, which could promote vimentin expression. However, further studies are required to explore the potential positive feedback loop between cVIM and vimentin.

HSCs, localized in the subendothelial space of Disse, only represent ~10% of all resident liver cells^42^. Hepatocytes are the main cells in the liver and damage to hepatocytes can elicit inflammation and fibrosis. Herein, cVIM was increased in isolated primary hepatocytes from CCl_4_ mice during liver fibrosis. Interestingly, cVIM was reduced in both isolated primary hepatocytes and primary HSCs from CCl_4_ mice after Ad-shcVIM treatment. In hepatocytes, loss of cVIM resulted in an increase in TGFBR1 as well as TGFBR2. In addition, cVIM downregulation promoted hepatocyte proliferation. Inhibiting cVIM also led to the suppression of collagen expression and TGF-β/Smads pathway. But these effects were almost inhibited by miR-9-5p and miR-122-5p inhibitor, which is similar with the results in HSCs after Ad-shcVIM treatment. Therefore, both hepatocytes and HSCs could be affected by Ad-shcVIM, which may be the reason why Ad-shcVIM contributed to the suppression of liver fibrosis in vivo. However, the effects of cVIM on other cells such as Kupffer were not studied and further studies are warranted to prove it. Notably, we also found that overexpression of cVIM in hepatocytes contributed to suppression of cell proliferation via cell cycle and had no effect on cell death (Supplementary Fig. 11), suggesting that cVIM inhibits hepatocyte proliferation without affecting hepatocyte viability.

Consistent with the results in vivo *and* in vitro, liver cVIM levels were found to be upreuglated in CHB patients as well as CHC patients, indicating that cVIM may be a potential biomarker in patients with liver fibrosis. Liver biopsy is the gold standard for assessing and/or monitoring liver injury severity. Compared with tissue biopsy, serum biomarkers are noninvasive and more easily accepted by patients. Next, serum cVIM level was examined in CHB patients and healthy controls. Our data showed that serum cVIM was increased in CHB patients. ROC curve analysis demonstrated that serum cVIM had a significant diagnostic value for liver fibrosis in patients with liver fibrosis. Interestingly, there was a positive correlation between serum cVIM and fibrosis scores. Serum cVIM may be a useful biomarker in patients with liver fibrosis and further studies with larger samples should be performed in the future.

In summary, we have demonstrated that loss of cVIM suppresses HSC activation, leading to inhibition of liver fibrosis. Our results support the involvement of a novel cVIM-miR-122-5p/miR-9-5p-TGF-β signaling cascade in liver fibrosis.

## Methods

### Mouse liver injury model

Male C57BL/6 J mice, aged 8 weeks, were given a 10% CCl_4_ solution in olive oil (7 μl × g^−1^ per mouse) through intraperitoneal injections every 2 weeks for a total of 8 weeks. To serve as a control, mice were given intraperitoneal injections of the same amount of olive oil at the corresponding time intervals. BDL was performed following previously established protocols^43^. In brief, mice were anesthetized using isoflurane and a midline incision was made to access the abdominal cavity. The common bile duct was identified and severed between non-absorbable thread ligatures. Sham-operated mice were employed as the experimental control group. Partial liver sections were subsequently stained with Masson solution. The areas exhibiting positive staining from the Masson stain were meticulously examined in sections obtained from all mice, with a minimum of 10 distinct fields per section. The remaining liver tissues were rapidly frozen in liquid nitrogen and preserved at −80 °C for subsequent analysis. The Experimental Animal Center of Wenzhou Medical University (WMU) supplied all animals used in this study. All experiments involving mice were conducted within the Experimental Animal Center and received approval from WMU’s Animal Care and Use Committee. We have complied with all relevant ethical regulations for animal use.

### Loss of cVIM in vivo

Twenty-four mice were randomly divided into four groups: the control group (*n* = 6) treated with olive oil, the model group (*n* = 6) treated with CCl_4_, the adenoviral vectors expressing scrambled shRNA (Ad-shCtrl) group (*n* = 6) treated with CCl_4_ and Ad-shCtrl, and the Ad-shcVIM group (*n* = 6) treated with CCl_4_ and Ad-shcVIM. Prior to the administration of CCl_4_, either Ad-shcVIM (1 × 10^9^ pfu per 100 μl) or Ad-shCtrl was injected via the tail vein 1 day in advance. Following a period of 4 weeks subsequent to CCl_4_ treatment, the mice were humanely euthanized.

### Isolation and culture of primary HSCs and Hepatocytes

After in situ perfusion of the liver with pronase and collagenase, primary HSCs were obtained using a discontinuous density gradient layering technique^44^. Subsequently, the isolated cells were inoculated and cultured. The purity of the HSC cultures was determined to be greater than 98% through immunocytochemical staining for α-SMA. On the first day following isolation, the examination of primary HSCs was conducted throughout the entirety of the experiments. The isolation process involved the utilization of a two-step collagenase perfusion technique^45^. Subsequently, the measurement of gene expression levels, specifically F4/80, CD32b, and CYP3A11, was performed using qRT-PCR. The purity of the hepatocytes was determined to exceed 95%.

### CircRNA microarray analysis

To examine liver circRNAs that were differentially expressed between CCl_4_-treated mice (*n* = 3) and healthy control mice (*n* = 3), we employed the Arraystar Mouse circRNA Array V2 (manufactured by KangChen Bio-tech in Shanghai, China). The measurement of total RNA from each sample was performed utilizing the NanoDrop ND-1000 spectrophotometer. The procedures for sample preparation and microarray hybridization followed the established protocols provided by Arraystar. In brief, the total RNA underwent digestion with RNAse R (Epicenter, Inc.) to eliminate linear RNA and enhance the presence of circular RNA. Next, the enhanced circular RNAs underwent amplification and transcription into fluorescent cRNA utilizing the random priming technique (Arraystar Super RNA Labeling Kit; Arraystar). Afterwards, the marked cRNAs were hybridized onto the Arraystar Mouse circRNA Array V2 (8x15K, Arraystar). The slides were washed and subsequently scanned using the Agilent Scanner G2505C. Agilent Feature Extraction software was used to evaluate the resulting images. Quantile normalization were conducted utilizing the “Limma” R package. Volcano Plot filtering was used to identify circRNAs that were expressed differently between the two groups. Hierarchical clustering was conducted to demonstrate the discernible patterns of circRNA expression across the samples.

### qRT-PCR

Cells and tissues were used to extract total RNA with the miRNeasy Mini Kit (Qiagen, Valencia, CA, USA). Afterwards, the RNA (50 ng) was reverse-transcribed using the ReverTra Ace qPCR RT kit (Toyobo, Osaka, Japan). The appropriate purification kits (Norgen, Thorold, Canada) were used to isolate cytoplasmic or nuclear cVIM from HSCs^46^. The qRT-PCR analysis was then conducted employing SYBR Green (Toyobo, Osaka, Japan) as a facilitator. The primer sequences used in the analysis can be found in Supplementary Table 2. Primers targeting Col1A1, α-SMA, and GAPDH were developed according to prior studies^47,48^. To assess the expression of 33 miRNAs, the TaqMan MicroRNA assay from Applied Biosystems in Foster City, CA was utilized. The GAPDH level was used to normalize the relative abundance of cVIM, mRNAs, and U6 snRNA to that of miRNAs. The 2^−⊿⊿Ct^ method was utilized to compute the levels of cVIM, mRNA, and miRNA expression.

### Western blot analysis

Tissues and cells were lysed using an ice-cold lysis buffer. The total protein content was determined, followed by separation through SDS-PAGE and subsequent analysis via western blot, following a previously established protocol^47^. Protein quantities were then normalized relative to the total amount of GAPDH present.

### Actinomycin D assay

The Actinomycin D assay was conducted following established protocols^49^. A total of 5 × 10^4^ cells was evenly distributed among 5 wells of 24-well plates. After 24 h, the cells were treated with actinomycin D at a concentration of 2 µg × ml^−1^ for durations of 0, 12, and 24 h. Subsequently, the cells were collected and the relative RNA levels of cVIM and mVIM were assessed using qRT-PCR.

### FISH

The double FISH assay was performed in cells as described previously with minor modifications^17^. Biotin-labeled probes specific for cVIM and Dig-labeled miR-122-5p and miR-9-5p probes were utilized for hybridization. The biotin-labeled probes emitted signals that were detected by utilizing Cy5-Streptavidin, whereas the signals emitted by Dig-labeled miR-122-5p and miR-9-5p probes were detected by employing a tyramide-conjugated Alexa 488 fluorochrome TSA kit. Furthermore, the cell nuclei were stained with DAPI and the acquired images were captured using a confocal microscope produced by Leica Microsystems in Mannheim, Germany.

### CCK-8 assay

Cells were seeded in 96-well plates at a density of 1 × 10^3^ cells per well and incubated for 24 h. Subsequently, transduction with cVIM siRNA-1 or cVIM siRNA-2 was performed for 24, 48, or 72 h. Cell proliferation was assessed utilizing the CCK-8 kit (Dojindo, Kumamoto, Japan). Absorbance was read at 450 nm on a microplate reader.

### Immunofluorescence microscopy

Cells were initially placed on 18 mm cover glasses and then treated with a solution of acetic acid: ethanol (1:3) for a duration of 5 min at a temperature of −20 °C. To prevent nonspecific binding, a mixture of goat serum and PBS (5%) was applied for 1 h at room temperature. Following this, the cells were exposed to primary antibodies targeting mouse α-SMA or type I collagen, and subsequently treated with Alexa Fluor 594-labeled rabbit anti-mouse IgG (diluted at a ratio of 1:50; Dianova). Nuclei were stained with DAPI. The slides were washed and covered with DABCO (Sigma-Aldrich, St. Louis, MO, USA), and examined using a confocal laser scanning microscope (Olympus, Tokyo, Japan) at a wavelength of 568 nm.

### RIP assay

The RIP study was conducted using the EZ-Magna RIP Kit from Millipore. In short, primary HSCs were lysed using a comprehensive RIP lysis buffer. Then, they were incubated with RIP buffer that had magnetic beads attached to anti-Ago2 antibody (Abcam). Isotype-matched IgG was employed as a negative control. Afterward, the samples were incubated with Proteinase k, and the isolated immunoprecipitated RNA was used for qRT-PCR analysis in order to assess the levels of cVIM in the precipitates.

### CircRIP

Biotin-labeled cVIM and control probes were synthesized by Sangon Biotech. The circRIP assay was performed as specified earlier^48^. In brief, the supernatant of cVIM-overexpressing cells was added to a probe-M280 streptavidin Dynabead (Invitrogen) mixture and further incubated at 30 °C for 12 h. The probe-Dynabead-circRNA mixture was washed and incubated with 200 µl lysis buffer and proteinase K. TRIzol Reagent (Invitrogen) was used to extracted RNA.

### Pull-down assay with Bio-miR-122-5p/ Bio-miR-9-5p

As described earlier, HSCs were transfected with Bio-miR-122-5p-Wt/Bio-miR-9-5p-Wt, Bio-miR-122-5p-Mut/Bio-miR-9-5p-Wt or Bio-microRNA negative control (Bio-miR-NC) for 48 h^50,51^. After 4 h at 4 °C with streptavidin-coated magnetic beads (Life Technologies), lysates were washed with lysis buffer, low-salt buffer and high-salt buffer. RNA extraction was performed using TRIzol Reagent (Invitrogen), and the expression of cVIM was determined using qRT-PCR.

### Luciferase reporter assay

As in our previous study, pmirGLO-cVIM was cotransfected with the predicted miRNAs or miR-NC into HEK293T cells^47^. After 48 h of transfection, the relative luciferase activity was normalized to that of Renilla luciferase.

### ChIP assay

Cell lysates were sonicated in order to produce 200–300 bp chromatin fragments and immunoprecipitated with a Sp1-specific antibody (Millipore) or IgG control. qRT-PCR was used to ascertained precipitated chromatin DNA.

### Immunohistochemistry

After deparaffinization, hydration, and antigen retrieval, 3 μm thick sections from liver tissues were incubated overnight at 4 °C with a primary antibody against α-SMA (1:100). Subsequently, sections were incubated with biotinylated secondary antibody. Quantitative analysis was performed on five fields for each liver slice.

### Signaling pathway assay

The Cignal Finder Reporter Array (Qiagen) was used to identify the relevant signaling pathways. Briefly, cVIM cDNA was amplified and inserted into the firefly luciferase plasmid. TOP-flash or FOP-flash (Millipore) and pRL-TK plasmid were cotransfected into cells (1 × 10^5^) in 24-well plates. Activities of both Renilla and firefly luciferase reporters determined after 48 h with the aid of a dual-luciferase reporter gene assay system (Promega). The determination of the TOP-Flash reporter activity was based on the relative ratio of firefly luciferase to Renilla luciferase activity.

### Adenoviral transduction

Pre-experimentation determined that the optimal multiplicity of infection for adenoviral vectors expressing cVIM (Ad-cVIM) or a control scrambled sequence (Ad-Ctrl) infection of primary HSC was 100. Transfection was performed at 60% cell confluence and after 12 h of transfection, the complete medium was changed. Cells were collected for subsequent studies.

### Statistics and reproducibility

Data from at least three independent experiments were expressed as the means ± SD. Differences among multiple groups were evaluated using one-way analysis of variance and those between groups using Student’s *t*-test. The significance of serum cVIM levels was determined using the Mann–Whitney test. ROC curves were generated to assess the diagnostic potential of cVIM by calculating the AUC, sensitivity and specificity according to standard formulae. Data were considered significant at *P* < 0.05. All statistical analyses were performed using SPSS software (version 13; SPSS, Chicago, IL).

### Reporting summary

Further information on research design is available in the Nature Portfolio Reporting Summary linked to this article.

### Supplementary information

Supplementary Information

Description of Additional Supplementary Files

Supplementary Data 1

Reporting Summary

## Data Availability

Supplementary Data 1 contains the source data for the graphs in the main figures. Supplementary Fig. 12 contains the original uncropped blot/gel images of the main figures. The other data supporting the findings of this study are available from the corresponding author upon reasonable request.
