# Supplementary material for: Circular RNA cVIM promotes hepatic stellate cell activation in liver fibrosis via miR-122-5p/miR-9-5p-mediated TGF-β signaling cascade
Source: Commun Biol. 2024 Jan 19;7:113. doi: 10.1038/s42003-024-05797-3 (PMC10798957; doi:10.1038/s42003-024-05797-3)
Supplement: Supplementary file 1 — Supplementary Information [file 42003_2024_5797_MOESM1_ESM.pdf]

**Supporting Information**

**Supplementary materials and methods**

**Patients and samples**

Overall, 15 healthy controls, 15 chronic hepatitis B (CHB) patients and 15 CHC patients undergoing liver biopsy from the First Affiliated Hospital of Wenzhou Medical University (FAHWMU) from January 2011 to June 2013 were selected. Serum samples were obtained from patients attending the FAHWMU from January 2011 to December 2015. In total, 110 therapy-naive patients who had undergone liver biopsy for staging and grading of CHB as well as 110 healthy controls (normal liver biochemistry, no history of liver disease, alcohol abuse or viral hepatitis) were enrolled (Table S1). Inclusion criteria were as follows: CHB defined by detectable serum HB antigen and serum HBV DNA for more than six months. Exclusion criteria were as follows: (i) patient ages <16 years, (ii) co-infection with human immunodeficiency virus (HIV), (iii) coexistence of liver injury caused by other etiologies, including hepatitis C virus (HCV) infection, drug intake, alcohol consumption and auto-immune hepatitis, (iv) severe systematic diseases, (v) pregnancy and lactation. Demographic and clinical information was additionally obtained from all patients. Our study was approved by the Ethics Committee of the FAHWMU and informed consent for use of liver or blood samples obtained from all participants. All ethical regulations relevant to human research participants were followed.

**Liver histology**

A 16-gauge Menghini needle was used to performed liver biopsy. Each liver specimen, at least in 2.0 cm, was advised by physicians in care. Samples were collected for hematoxylin-eosin staining, and experienced hepatopathologists reviewed the results. Additionally, a minimum of

8-10 portal tracts in samples was necessary for patient admission. Fibrosis stage (F0 = no fibrosis – F6 = cirrhosis) was assessed using the Ishak scoring system.

**Blood sampling**

At the time of liver biopsy, blood samples of each patient were collected. Samples were centrifuged at 3400 g for 7 min at room temperature and at 12,000 *g* for 10 min at 4°C to remove the remaining cells. They were then stored at -80°C for further processing.

**Virology**

Serum HBV DNA was detected using the Artus HBV QS-RGQ Kit (Qiagen) with a lower detection limit of 10.2 IU/ml. Roche Modular E170 Immunoassay Analyzer (Roche) was uesd to quantified HBsAg, HBeAg and antibodies against HBsAg, HBeAg and hepatitis B core antigen.

**Supplementary Tab.1: Characteristics of CHB patients and healthy subjects**

| | | CHB patients (n=110) | healthy subjects (n=110) |
|---|---|---|---|
| Age (years) | Mean±SD | 41.5±9.3 | 42.2±10.1 |
| Sex (n) | Male | 63(57.2%) | 61 (55.4%) |
| | Female | 47 (42.8%) | 49 (44.6%) |
| ALT (U/L) | Mean±SD | 115.3±96.1 | |
| Liver HBV DNA (IU/cells) | Mean±SD | 2.569±4.766 | |
| Serum cVIM | Mean±SD | 1.869±0.430 | |
| HBeAg | + | 53 (48.1%) | |
| | - | 57 (51.9%) | |
| Fibrosis (n) | F0 | 7 (6.4%) | |
| | F1 | 18 (16.4%) | |
| | F2 | 15 (13.6%) | |
| | F3 | 16 (14.5%) | |
| | F4 | 13 (11.8%) | |
| | F5 | 27 (24.5%) | |
| | F6 | 14 (12.7%) | |

**Supplementary Tab.2: Primers used in this study.**

| Primer Name | Sequence (5'-3') |
|---|---|
| Mouse | |
| cVIM-F | ACGGTTGAGACCAGAGATGG |
| cVIM-R | AGTGAGGTCAGGCTTGGAAA |
| cANRIL-F | CCTCCTCATGTGGAATCACC |
| cANRIL-R | TTCAAGGGTCAGCCTCATCT |
| TGFBR1-F | AAAGCAGTCAGCTGGCCTTG |
| TGFBR1-R | AGGTGGTGCCCTCTGAAATG |
| TGFBR2-F | TGGCTTCGAACACCATGGAA |
| TGFBR2-R | TAGAGGGCGGTGAACAACAG |
| NOX4-F | TGGCCAACGAAGGGGTTAAA |
| NOX4-R | TCGCCCAACATTTGGTGAATG |
| KLF6-F | TGTGGGGTCAAATACAGGGGAA |
| KLF6-R | AAGGACTTTTCACCCGTTCGTTCA |
| TGFβ1-F | ACTGCAAGTCAGAGACGTGG |
| TGFβ1-R | GGAATAGGGGCGTCTGAGGA |
| SRF-F | AGTTGGGGTAGGGTGTCACT |
| SRF-R | CCCCATGAAACGTAGGCTGT |
| FN1-F | GGTCACCCTGTTCTGCTTCA |
| FN1-R | TGTCTGGGTGACTTTCCTGC |
| Sp1-F | CCACCATGAGCGACCAAGAT |
| Sp1-R | CGCTACCCCCATTATTGCCA |
| Col1A1-F | CGATGGATTCCCGTTCGAGT |
| Col1A1-R | GAGGCCTCGGTGGACATTAG |
| α-SMA-F | TCTTCCAGCCATCTTTCATTGGGAT |
| α-SMA-R | CCTGTTTTGGCTCCCTATGTCT |
| Vimentin-F | AGACCAGAGATGGACAGGTGA |
| Vimentin-R | CTGGTACTGCACTGTTGCAC |
| TIMP1-F | GCGGTTCTGGGACTTGTGGGCATA |
| TIMP1-R | GCCCCCTTTGCATCTCTGGCATC |
| MMP2-F | ACCTGGATGCCGTCGTGGACCTG |
| MMP2-R | CGCCAGGCTGCTTCACATCCTTC |
| IL-6-F | ACAACCACGGCCTTCCCTACTT |
| IL-6-R | CACGATTTCCCAGAGAACATGTG |
| TGF-β1-F | GCCCTGGATACCAACTATTGCTTCA |
| TGF-β1-R | CAGAAGTTGGCATGGT |
| TINCR-F | CAAGGTACACCTAGCCGACG |
| TINCR-R | TTCTGCGCCAAGAGCTTACA |
| GAPDH-F | AGGAGAGTGTTTCCTCGTCC |
| GAPDH-R | TGAGGTCAATGAAGGGGTCG |
| U6-F | GAAGATTTAGCATGGCCCCTGC |
| U6-R | CAGTGCAGGGTCCGAGGT |
| cVIM-siRNA-1 | TGCCCTTAAAGGCACTAACGAGT |
| cVIM-siRNA-2 | AGGATGAGATCCAAAACATGAAG |

| | |
|---|---|
| Human | |
| hsa_circ_0000221-F | TGTCGATGTAGTTGGCGAAG |
| hsa_circ_0000221-R | ACCTCAACGAGAAGGTGGAG |
| hsa_circ_0007939-F | GGTCAAAGGTATGGCATTGG |
| hsa_circ_0007939-R | ACTGCTCTTTCCCTGGCTTT |
| hsa_circ_0017865-F | CGTCACCTTCGTGAATACCA |
| hsa_circ_0017865-R | AAACTTCTGCAGCCTTTGGA |
| hsa_circ_0017866-F | TTCCAGCAAGTATCCAACCA |
| hsa_circ_0017866-R | AAAGCCTGTCTTTGCTCGAA |
| hsa_circ_0017867-F | AGATGGCCCTTGACATTGAG |
| hsa_circ_0017867-R | GGAGAAGAGGCGAACGAG |
| hsa_circ_0017868-F | GGATTCACTCCCTCTGGTTG |
| hsa_circ_0017868-R | GGAGAAGAGGCGAACGAG |
| hsa_circ_0017869-F | TTCCAGCAAGTATCCAACCA |
| hsa_circ_0017869-R | GGAGAAGAGGCGAACGAG |
| hsa_circ_0017870-F | TCTGGATTCACTCCCTCTGG |
| hsa_circ_0017870-R | CGCATTGTCAACATCCTGTC |
| hsa_circ_0017871-F | TTCCAGCAAGTATCCAACCA |
| hsa_circ_0017871-R | CGCATTGTCAACATCCTGTC |
| hsa_circ_0017872-F | TGGATTCACTCCCTCTGGTT |
| hsa_circ_0017872-R | ATTCCACTTTGCGTTCAAGG |
| hsa_circ_0017873-F | TCCAGCAAGTATCCAACCAA |
| hsa_circ_0017873-R | CCTCTTCGTGGAGTTTCTTCA |
| hsa_circ_0017874-F | GTACCGGAGACAGGTGCAGT |
| hsa_circ_0017874-R | GGCTTGGAAACATCCACATC |
| hsa_circ_0017875-F | AGATGGCCCTTGACATTGAG |
| hsa_circ_0017875-R | GGCTTGGAAACATCCACATC |
| hsa_circ_0017876-F | TCTGGATTCACTCCCTCTGG |
| hsa_circ_0017876-R | GTGAGGTCAGGCTTGGAAAC |
| hsa_circ_0017877-F | TTCCAGCAAGTATCCAACCA |
| hsa_circ_0017877-R | GGCTTGGAAACATCCACATC |
| hsa_circ_0017878-F | TCCAGCAAGTATCCAACCAA |
| hsa_circ_0017878-R | CTGCACCTGTCTCCGGTACT |
| hsa_circ_0017879-F | CCTACAGGAAGCTGCTGGAA |
| hsa_circ_0017879-R | GCTTCAACGGCAAAGTTCTC |
| hsa_circ_0017880-F | TCTGGATTCACTCCCTCTGG |
| hsa_circ_0017880-R | AACGGCAAAGTTCTCTTCCA |
| hsa_circ_0017881-F | TCCAGCAAGTATCCAACCAA |
| hsa_circ_0017881-R | AGGCGGCCAATAGTGTCTT |
| hsa_circ_0017882-F | TCCAGCAAGTATCCAACCAAC |
| hsa_circ_0017882-R | CCAGAGGGAGTGAATCCAGA |
| hsa_circ_0017883-F | CGGTTGAAACTAGAGATGGACA |
| hsa_circ_0017883-R | TGAGTGGGTATCAACCAGAGG |
| hsa_circ_0017884-F | TTCCAGCAAGTATCCAACCA |
| hsa_circ_0017884-R | ATTGCTGCACTGAGTGTGTG |
| hsa_circ_0093230-F | GCAGGAGGAGATGCTTCAGA |

| | |
|---|---|
| hsa_circ_0093230-R | ACTGGCTCCCGGAGAAGAG |
| hsa_circ_0093232-F | ACCTCTACGAGGAGGAGATGC |
| hsa_circ_0093232-R | GCAGGATCTTATTCTGCTGCTC |
| hsa_circ_0093233-F | ACCTCTACGAGGAGGAGATGC |
| hsa_circ_0093233-R | TGTCGATGTAGTTGGCGAAG |

**Supplementary Tab.3: Up-regulated or down-regulated circRNAs in liver fibrosis**

| circRNA | Regulation | GeneSymbol | P-value |
|---|---|---|---|
| mmu_circRNA_29981 | up | App | 0.049175159 |
| mmu_circRNA_012164 | up | Hist1h1c | 0.002970874 |
| mmu_circRNA_20588 | up | Pam | 0.014034001 |
| mmu_circRNA_37780 | up | Syf2 | 0.024078973 |
| mmu_circRNA_007217 | up | Hist1h1c | 0.003261803 |
| mmu_circRNA_40537 | up | Antxr1 | 0.033347472 |
| mmu_circRNA_23791 | up | Spag5 | 0.0381213 |
| mmu_circRNA_36840 | up | Svep1 | 0.034132532 |
| mmu_circRNA_018777 | up | Col3a1 | 0.009699984 |
| mmu_circRNA_26326 | up | Sema4d | 0.016765115 |
| mmu_circRNA_34451 | up | Fbn1 | 0.010741952 |
| mmu_circRNA_23124 | up | Rtn4 | 0.049617416 |
| mmu_circRNA_29984 | up | Ltn1 | 0.040634076 |
| mmu_circRNA_37916 | up | Pgd | 0.027174359 |
| mmu_circRNA_015902 | up | Clip2 | 0.005204066 |
| mmu_circRNA_24182 | up | Acly | 0.000201364 |
| mmu_circRNA_19118 | up | Samd4 | 0.048250246 |
| mmu_circRNA_37328 | up | Dhcr24 | 0.028201056 |
| mmu_circRNA_30668 | up | Adgre1 | 0.003752486 |
| mmu_circRNA_19794 | up | Lmbrd1 | 0.043492598 |
| mmu_circRNA_19982 | up | Map4k4 | 0.042358766 |
| mmu_circRNA_27519 | up | Samd4 | 0.038562935 |
| mmu_circRNA_19346 | up | Pgd | 0.037615846 |
| mmu_circRNA_34309 | up | Rtf1 | 0.004720457 |
| mmu_circRNA_32665 | up | Btrc | 0.031973352 |
| mmu_circRNA_39081 | up | Sparcl1 | 0.0101094 |
| mmu_circRNA_34779 | up | Tpx2 | 0.021333323 |
| mmu_circRNA_29154 | up | Ppl | 0.00153531 |
| mmu_circRNA_28239 | up | Npr3 | 0.049582734 |
| mmu_circRNA_005305 | up | Samd4 | 0.049573841 |
| mmu_circRNA_45890 | up | Rps6ka3 | 0.04247312 |
| mmu_circRNA_26413 | up | Ntrk2 | 0.041744953 |
| mmu_circRNA_39653 | up | Baiap2l1 | 0.002078411 |
| mmu_circRNA_22197 | up | Bicc1 | 0.037300025 |
| mmu_circRNA_017702 | up | Slc35f5 | 0.042870363 |
| mmu_circRNA_44559 | up | Myo1e | 0.005579929 |
| mmu_circRNA_36691 | up | Unc13b | 0.005411979 |
| mmu_circRNA_34279 | up | Knstrn | 0.034316795 |
| mmu_circRNA_000298 | up | Ect2 | 0.001133466 |
| mmu_circRNA_32994 | up | Vim | 0.020330809 |
| mmu_circRNA_34544 | up | Sirpa | 0.011532885 |
| mmu_circRNA_011784 | up | Arhgap10 | 0.008056417 |
| mmu_circRNA_45392 | up | Rp2h | 0.013447414 |
| mmu_circRNA_30260 | up | Smoc2 | 0.001386797 |
| mmu_circRNA_006940 | down | 9130011E15Rik | 0.043846956 |
| mmu_circRNA_25777 | down | Serpina4-ps1 | 0.017909055 |
| mmu_circRNA_21916 | down | Rtn4ip1 | 0.049761176 |

| | | | |
|---|---|---|---|
| mmu_circRNA_21120 | down | Tnr | 0.047860526 |
| mmu_circRNA_28144 | down | Ghr | 0.032418353 |
| mmu_circRNA_29063 | down | Fam186b | 0.004624547 |
| mmu_circRNA_28834 | down | Slc25a17 | 0.039413973 |
| mmu_circRNA_22878 | down | Camk2b | 0.043426037 |
| mmu_circRNA_21911 | down | Sobp | 0.025312431 |
| mmu_circRNA_25732 | down | Atxn3 | 0.046665629 |
| mmu_circRNA_39652 | down | Lmtk2 | 0.038077759 |
| mmu_circRNA_37243 | down | Raver2 | 0.043913776 |
| mmu_circRNA_39156 | down | Ep400 | 0.002552742 |
| mmu_circRNA_32345 | down | Kank1 | 0.016208401 |
| mmu_circRNA_42397 | down | Lhpp | 0.018030244 |
| mmu_circRNA_42398 | down | Lhpp | 0.007353568 |
| mmu_circRNA_26977 | down | Ppap2a | 0.037184896 |
| mmu_circRNA_22784 | down | Tmem194 | 0.021444162 |
| mmu_circRNA_36581 | down | Bach2 | 0.046418701 |
| mmu_circRNA_34113 | down | Slc1a2 | 0.000341918 |
| mmu_circRNA_25382 | down | Gphn | 0.02254617 |
| mmu_circRNA_41020 | down | Slco1b2 | 0.006157182 |
| mmu_circRNA_38486 | down | Rgs12 | 0.023791373 |
| mmu_circRNA_31987 | down | Dym | 0.043242595 |
| mmu_circRNA_36004 | down | Slc30a7 | 0.047274749 |
| mmu_circRNA_32003 | down | Pias2 | 0.000185612 |
| mmu_circRNA_42780 | down | Dctn6 | 0.005768733 |

**Supplementary Tab.4: The target miRNAs of cVIM predicted by miRanda software.**

| miRNA | Max Score | Max Energy | Binding position In cVIM | Predicted by RNAhybrid |
|---|---|---|---|---|
| mmu-miR-30a-5p | 103 | -21.66 | 40,128 | Yes |
| mmu-miR-125a-3p | 109 | -26.37 | 57,179 | Yes |
| mmu-miR-9-5p | 97 | -20.27 | 611 | Yes |
| mmu-miR-149-3p | 98 | -24.89 | 520,149, 568,56 | Yes |
| mmu-miR-187-3p | 96 | -24.4 | 342,616,179,115 | Yes |
| mmu-miR-191-3p | 99 | -23.01 | 231,143 | Yes |
| mmu-miR-200b-5p | 95 | -24.46 | 73,428,294 | Yes |
| mmu-miR-143-5p | 98 | -29.24 | 236,98 | Yes |
| mmu-miR-23a-5p | 95 | -22.73 | 466,564,1 | Yes |
| mmu-miR-26b-3p | 101 | -24.94 | 488,222 | Yes |
| mmu-miR-93-3p | 96 | -20.79 | 479 | Yes |
| mmu-miR-34a-5p | 87 | -26.41 | 167,97 | Yes |
| mmu-miR-326-5p | 97 | -26.33 | 414,272,54,96 | Yes |
| mmu-miR-328-3p | 103 | -32.07 | 66,520,560,166,5 | Yes |
| mmu-miR-330-3p | 89 | -23.59 | 73,172 | Yes |
| mmu-miR-122-5p | 97 | -20.98 | 356 | Yes |
| mmu-miR-337-5p | 98 | -25.57 | 73,596 | Yes |
| mmu-miR-341-5p | 93 | -23.25 | 352,288 | Yes |
| mmu-miR-345-3p | 93 | -28.44 | 12,207,129 | Yes |
| mmu-miR-346-3p | 101 | -30.48 | 505,2,204 | Yes |
| mmu-miR-107-3p | 97 | -22.24 | 467 | Yes |
| mmu-miR-17-3p | 110 | -24.12 | 59,242 | Yes |
| mmu-miR-25-5p | 93 | -22.73 | 152 | Yes |
| mmu-miR-212-5p | 87 | -21.90 | 477 | Yes |
| mmu-miR-320-5p | 97 | -23.57 | 491,123 | Yes |
| mmu-miR-26a-2-3p | 94 | -21.47 | 227 | Yes |
| mmu-miR-29b-2-5p | 101 | -20.51 | 608,404 | Yes |
| mmu-miR-125b-1-3p | 102 | -24.85 | 57,232,116,1,169 | Yes |
| mmu-miR-217-5p | 90 | -20.61 | 247 | Yes |
| mmu-miR-378a-5p | 96 | -26.57 | 227,13,477,201 | Yes |
| mmu-miR-381-3p | 100 | -22.24 | 323 | Yes |
| mmu-miR-215-3p | 103 | -22.17 | 263 | Yes |
| mmu-miR-196b-5p | 106 | -24.29 | 472,98,236,286 | Yes |
| mmu-miR-412-5p | 99 | -22.31 | 566 | No |
| mmu-miR-434-3p | 103 | -25.66 | 133 | No |
| mmu-miR-465a-3p | 97 | -20.31 | 439 | No |
| mmu-miR-540-3p | 98 | -24.08 | 506,353 | No |
| mmu-miR-423-5p | 99 | -24.36 | 507,153,63,563 | No |
| mmu-miR-681 | 101 | -25.57 | 100,165 | No |
| mmu-miR-1298-3p | 99 | -21.02 | 22 | No |
| mmu-miR-764-3p | 100 | -24.73 | 354 | No |
| mmu-miR-652-5p | 103 | -24.27 | 562 | No |
| mmu-miR-490-3p | 98 | -25.20 | 557 | No |
| mmu-miR-693-3p | 111 | -22.00 | 468,163,94,247 | No |
| mmu-miR-146b-3p | 107 | -23.92 | 285,187,258 | No |
| mmu-miR-698-5p | 100 | -23.18 | 459,569 | No |

| | | | | |
|---|---|---|---|---|
| mmu-miR-717 | 101 | -21.47 | 502 | No |
| mmu-miR-3099-3p | 97 | -24.89 | 521,163,66 | No |
| mmu-miR-344d-1-5p | 103 | -21.54 | 100,352,6 | No |
| mmu-miR-676-3p | 97 | -24.61 | 116 | No |
| mmu-miR-615-3p | 103 | -27.21 | 7,612 | No |
| mmu-miR-453 | 100 | -22.41 | 156,351 | No |
| mmu-miR-92b-3p | 98 | -27.08 | 231,299,134 | No |
| mmu-miR-873a-5p | 101 | -20.90 | 280 | No |
| mmu-miR-877-3p | 103 | -25.33 | 490,134 | No |
| mmu-miR-1894-5p | 98 | -22.13 | 481 | No |
| mmu-miR-1930-3p | 99 | -22.47 | 242,76 | No |
| mmu-miR-1231-5p | 98 | -25.51 | 511,67,25,601,171 | No |

**Supplementary Tab.5: TGF-β pathway-related genes which are the targets of miR-9-5p and miR-122-5p.**

| TGF-β pathway-related genes | miRNAs | The studies proving that they are the targets of the indicated miRNAs |
|---|---|---|
| TGFBR1 and TGFBR2 | miR-9-5p | Epigenetically-Regulated MicroRNA-9-5p Suppresses the Activation of Hepatic Stellate Cells via TGFBR1 and TGFBR2 [1] |
| TGFBR2 | miR-9-5p | Protective role for miR-9-5p in the fibrogenic transformation of human dermal fibroblasts [2] |
| TGFBR2 and NOX4 | miR-9-5p | miR-9-5p suppresses pro-fibrogenic transformation of fibroblasts and prevents organ fibrosis by targeting NOX4 and TGFBR2 [3] |
| KLF6 | miR-122-5p | NEAT1 accelerates the progression of liver fibrosis via regulation of microRNA-122 and Kruppel-like factor 6 [4] |
| TGF-β1 | miR-122 | microRNA-122 down-regulation may play a role in severe myocardial fibrosis in human aortic stenosis through TGF-β1 up-regulation [5] |
| TGFBR2 | miR-122-5p | miR-24 and miR-122 Negatively Regulate the Transforming Growth Factor-β/Smad Signaling Pathway in Skeletal Muscle Fibrosis [6] |
| SRF and FN1 | miR-122-5p | Identification of a novel TGF-β-miR-122-fibronectin 1/serum response factor signaling cascade and its implication in hepatic fibrogenesis [7] |

**Supplementary Fig. 1 Expression of cVIM in primary HSCs and primary hepatocytes. a** Expressions of circRNA_39081, circRNA_32994 (cVIM) and circRNA_34779 in primary HSCs isolated from $CCl_4$-treated mice as well as healthy controls (n=6 per group). **b** cVIM was detected in primary HSCs and hepatocytes, which were isolated from healthy mice (n=6 per group). **(c and d**) cVIM was detected in primary HSCs (c) and hepatocytes (d), which were isolated from $CCl_4$-treared mice at different weeks (n=6 per group). Each value is the mean ± SD of six independent experiments. $^{**}P$<0.01 compared with the control.

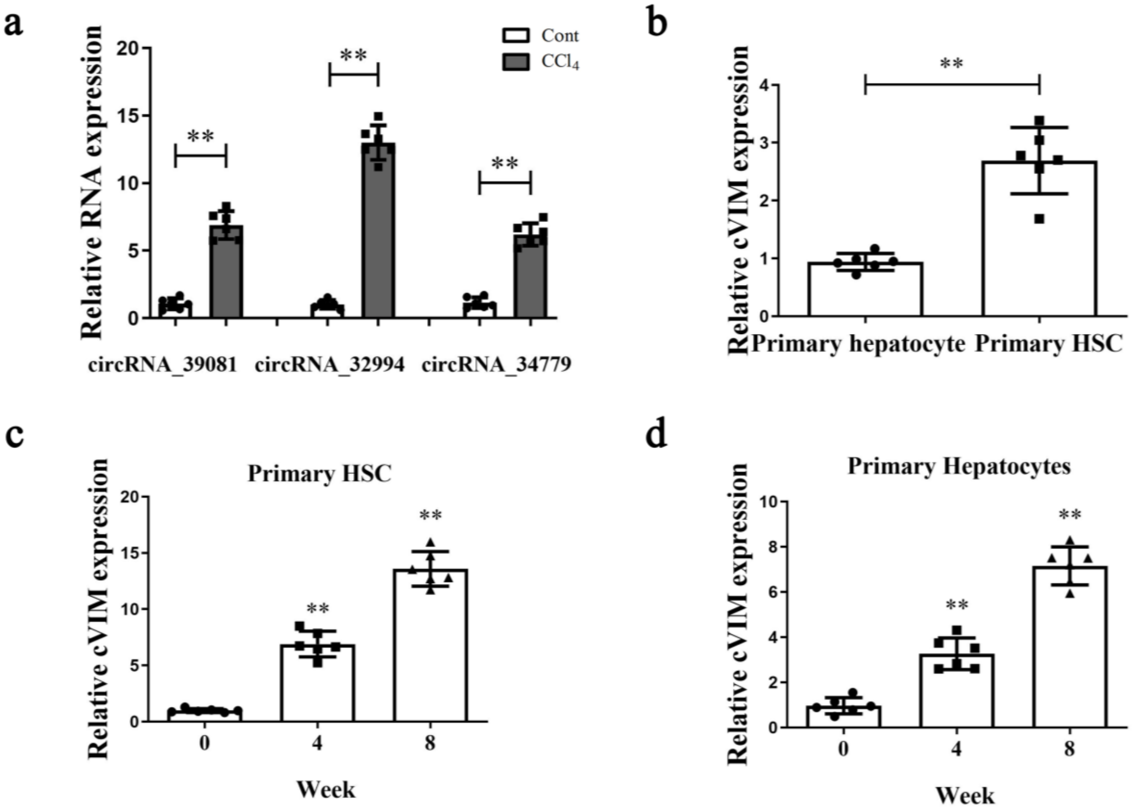

**Supplementary Fig. 2 Sanger sequencing showing the back-spliced events of cVIM.**

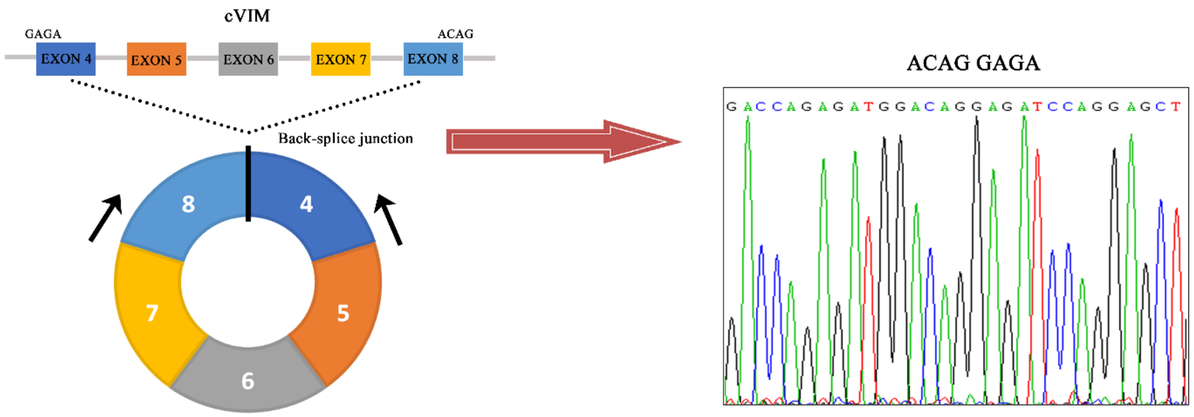

**Supplementary Fig. 3 Expression of cVIM *in vitro and in vivo* after cVIM knockdown treatment. a** Expression of cVIM in primary 1-day-old HSCs isolated from $CCl_4$-treated mice after cVIM siRNA treatment for 48 h (n=3 per group). **b** cVIM in the liver from $CCl_4$-treated mice after Ad-shcVIM treatment (n=6 per group). **(c and d**) cVIM in isolated primary HSCs (c) as well as primary hepatocytes (d) from $CCl_4$-treated mice after Ad-shcVIM treatment (n=6 per group). **e** Expression of mVIM after silencing of cVIM *in vitro* (n=3 per group) *and in vivo* (n=6 per group). **f** ECM markers in liver tissues of $CCl_4$-treated mouse (n=6 per group). **(g and h**) Profibrotic cytokines in HSCs (g) and in liver tissues (h) (n=6 per group). Each value is the mean ± SD of three independent experiments. **P*<0.05 and ***P*<0.01 compared with the control.

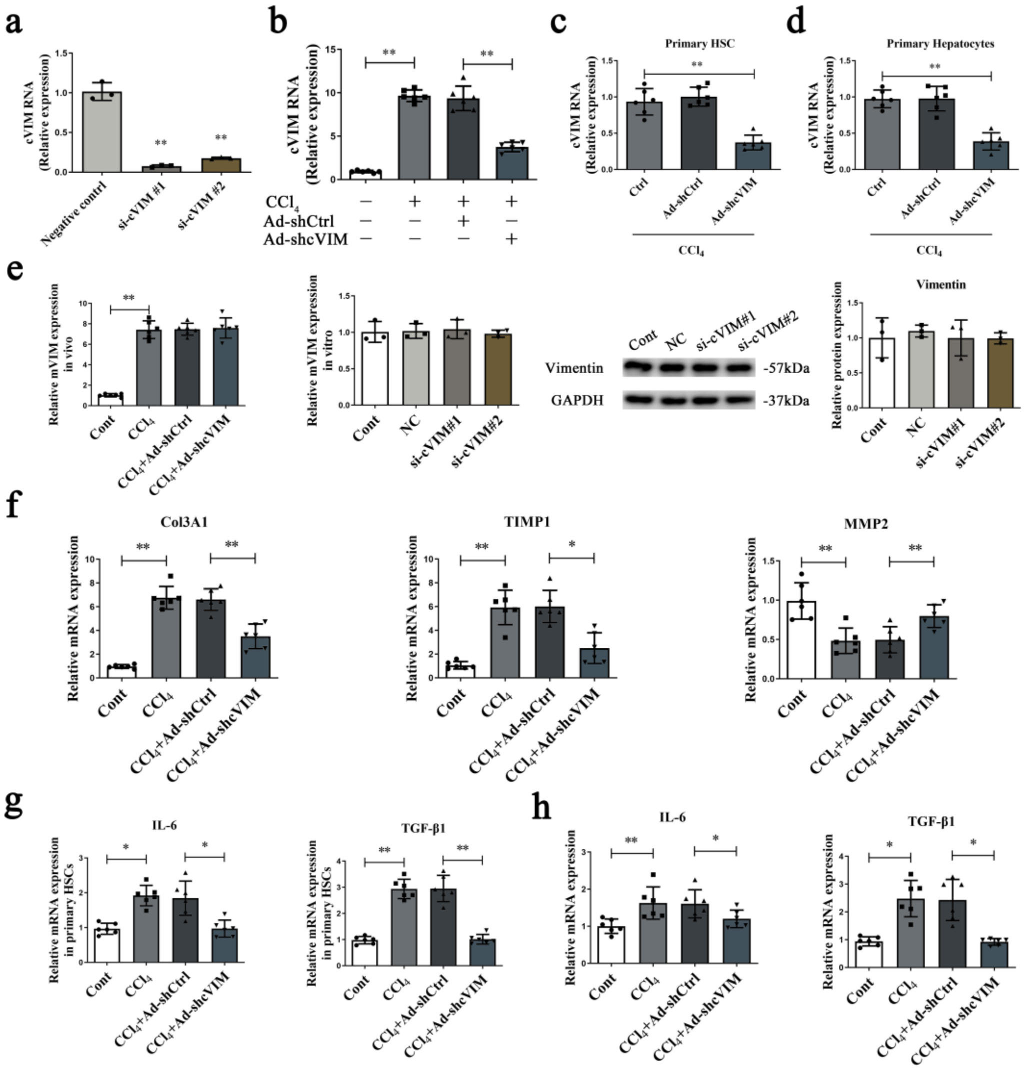

**Supplementary Fig.4 cVIM functions as a sponge for miR-122-5p and miR-9-5p. a** Co-localization between miR-122-5p/miR-9-5p and cVIM was observed by FISH in primary 1-day-old HSCs isolated from $CCl_4$-treated mice. Nuclei were stained with DAPI (n=3 per group). Scale bar, 25 μm. **b** qRT-PCR showed that cVIM did not change significantly upon miR-122-5p or miR-9-5p mimics/inhibitor in primary 1-day-old HSCs isolated from $CCl_4$-treated mice. qRT-PCR showed that miR-122-5p or miR-9-5p did not change significantly after overexpressing or silencing cVIM in primary 1-day-old HSCs isolated from $CCl_4$-treated mice (n=3 per group). **c** The expressions of miR-122-5p and miR-9-5p were decreased in the fibrotic livers (n=6 per group). Each value is the mean ± SD of three independent experiments. *$P$<0.05.

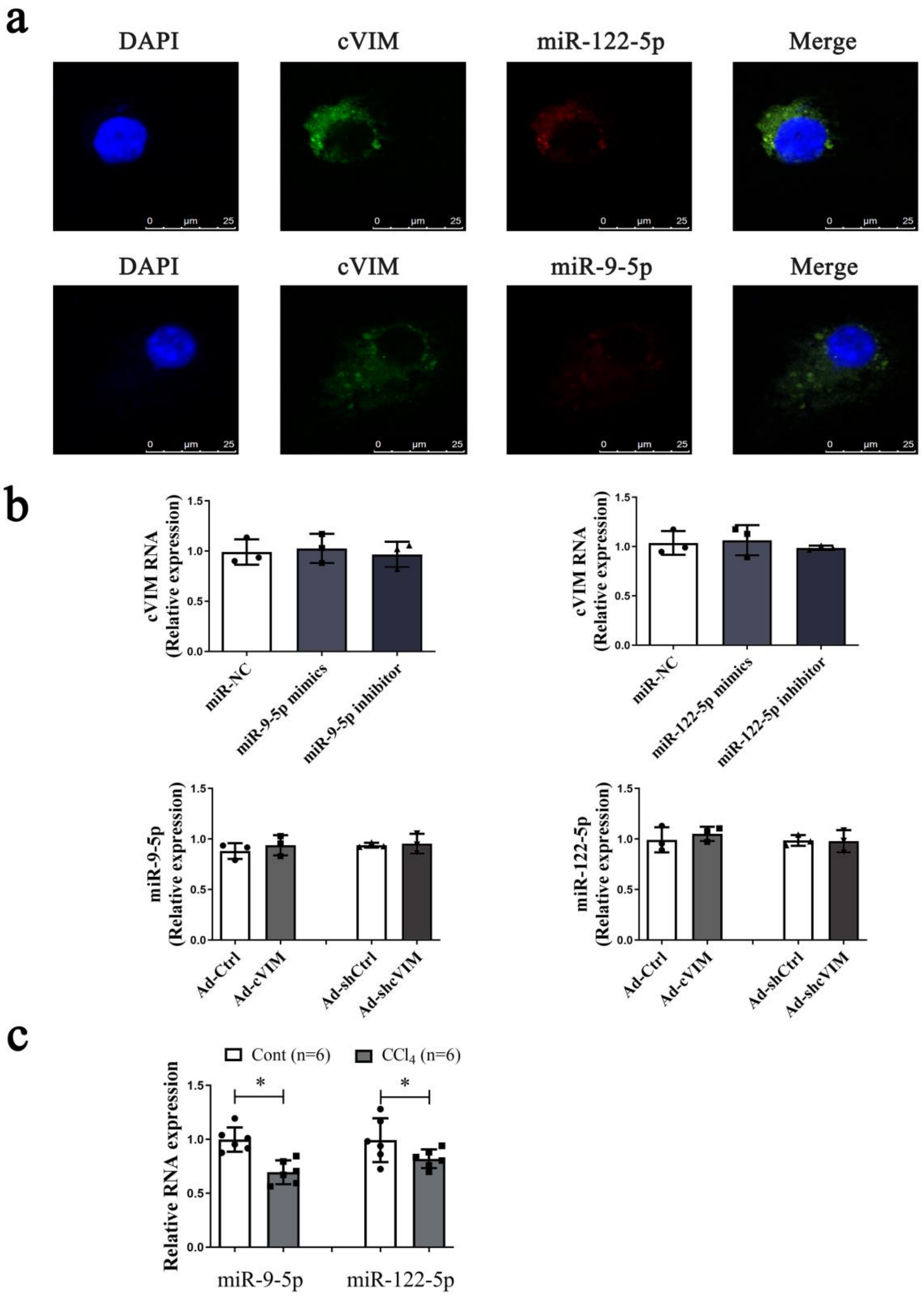
a
DAPI
cVIM
miR-122-5p
Merge
DAPI
cVIM
miR-9-5p
Merge
0 μm 25
b
cVIM RNA (Relative expression)
miR-NC
miR-9-5p mimics
miR-9-5p inhibitor
miR-122-5p mimics
miR-122-5p inhibitor
miR-9-5p (Relative expression)
miR-122-5p (Relative expression)
Ad-Ctrl
Ad-cVIM
Ad-shCtrl
Ad-shcVIM
c
Cont (n=6)
CCl4 (n=6)
Relative RNA expression
miR-9-5p
miR-122-5p

**Supplementary Fig.5 cVIM inhibits liver fibrosis through TGFBR1/TGFBR 2-mediated TGF-β pathway activation.** Primary 1-day-old HSCs isolated from $CCl_4$-treated mice were transduced with Ad-cVIM or Ad-shcVIM for 48 h. Cells were also transfected with miR-122-5p or miR-9-5p mimics for 24 h. **a** Signaling pathway reporter array was used for seeking the relative pathway associated with cVIM (n=3 per group). **b** qRT-PCR showed that the targets of miR-9-5p (TGFBR1, TGFBR2 and NOX4) were significantly downregulated upon overexpression of miR-9-5p, and the targets of miR-122-5p (KLF6, TGF-β1, TGFBR2, SRF and FN1) were significantly downregulated upon overexpression of miR-122-5p (n=3 per group). **c** The targets of miR-9-5p and miR-122-5p were examined in cells after overexpressing or silencing cVIM (n=3 per group). Each value is the mean ± SD of three independent experiments. $^{**}P<0.01$.

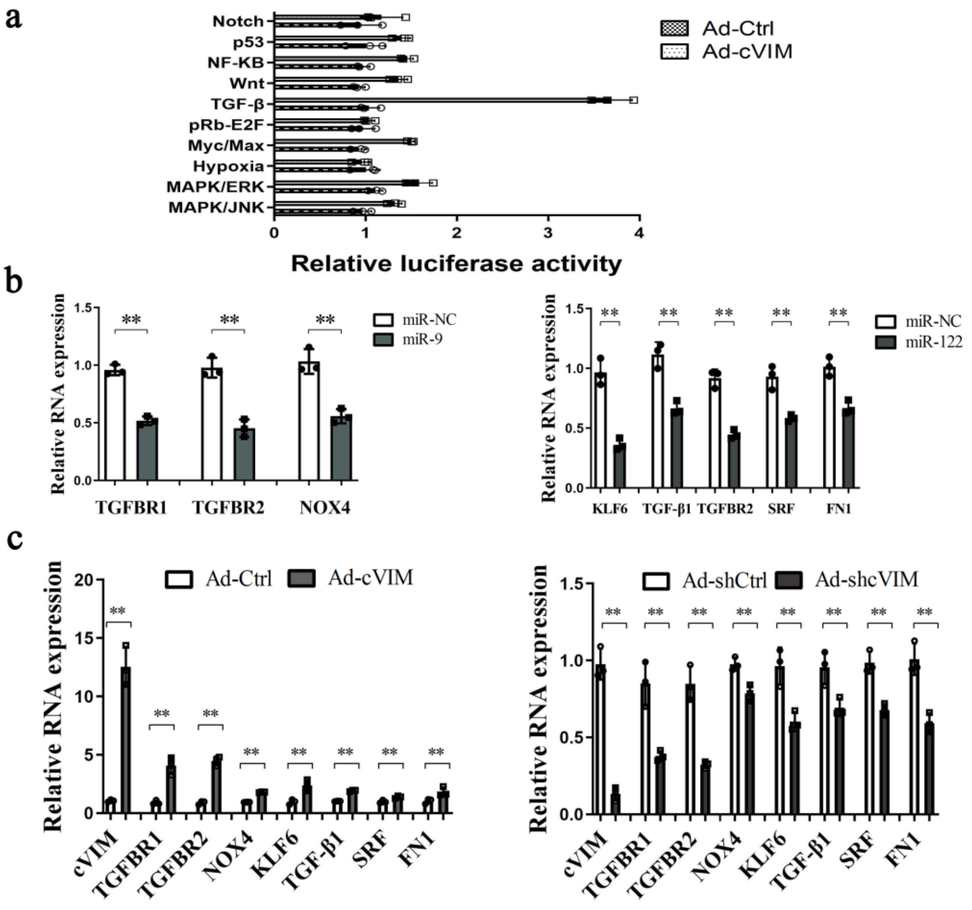

**Supplementary Fig.6 cVIM accelerates liver fibrosis progression via miR-122-5p/miR-9-5p-midaited TGF-β pathway.** Primary 1-day-old HSCs isolated from $CCl_4$-treated mice were transduced with Ad-cVIM for 48 h and then transfected with miR-122-5p/miR-9-5p mimics for additional 24 h. Moreover, cells were transduced with Ad-shcVIM for 48 h and then transfected with miR-122-5p/miR-9-5p inhibitor for additional 24 h. **a** Reduced TGFBR1 by miR-122-5p or miR-9-5p was blocked down by overexpression of cVIM (n=3 per group). **b** Reduced TGFBR2 by miR-122-5p or miR-9-5p was blocked down by overexpression of cVIM (n=3 per group). **c** Immunobolt analysis showed that miR-122-5p or miR-9-5p inhibitor inhibited the downregulation of p-smad2, α-SMA and type I collagen induced by cVIM knockdown (n=3 per group). Each value is the mean ± SD of three independent experiments. **P*<0.05, ***P*<0.01 and ****P*<0.001.

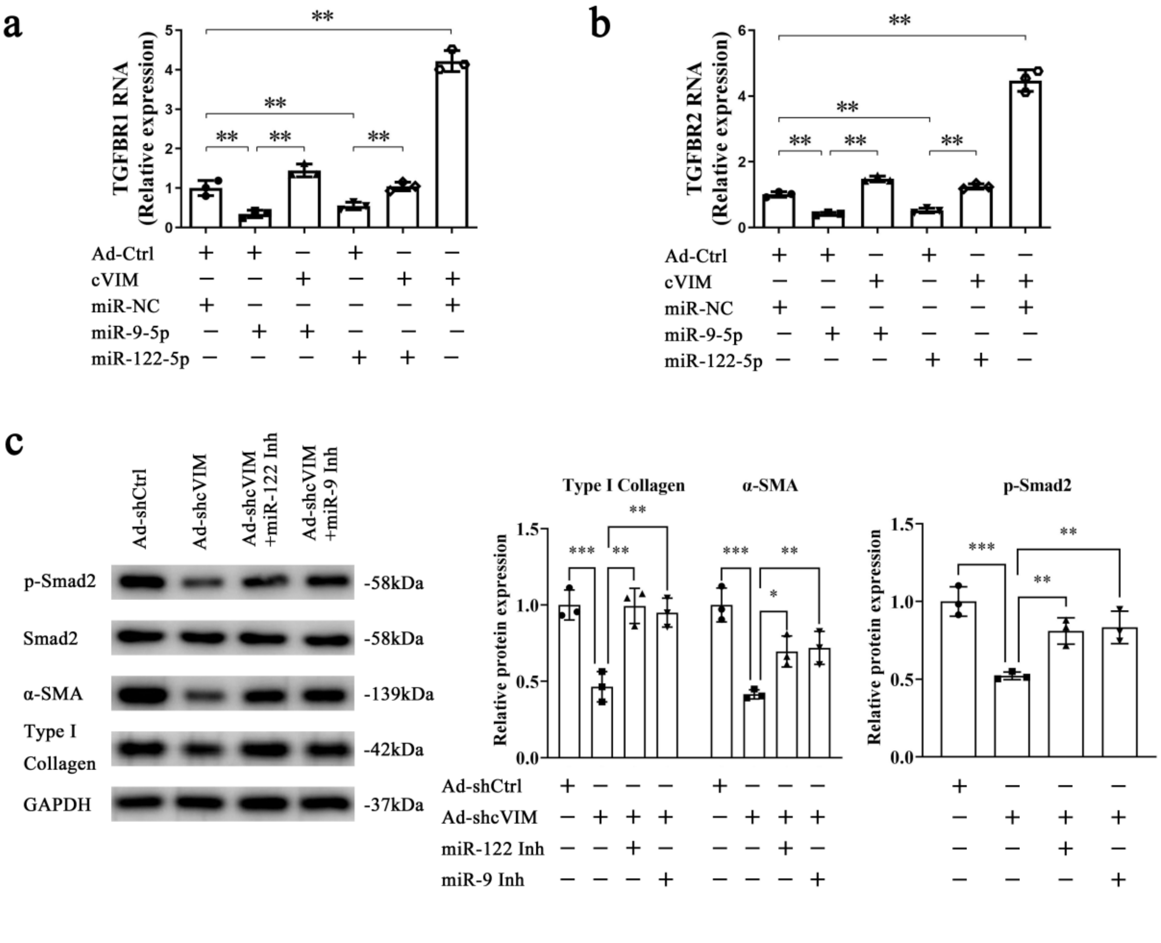

**Supplementary Fig.7 Overexpression of cVIM contributes to the expression of Sp1.** Primary 1-day-old HSCs isolated from $CCl_4$-treated mice were transduced with Ad-cVIM or Ad-shcVIM for 48 h. **a** Sp1 mRNA expression (n=3 per group). **b** Sp1 protein expression (n=3 per group). **c** Expression of Sp1 in activated HSCs isolated from $CCl_4$-treated mice (n=6 per group). **d** Expression of Sp1 in CHB patients with liver fibrosis (n=15 per group). Each value is the mean ± SD of three independent experiments. *$P$<0.05 and **$P$<0.01.

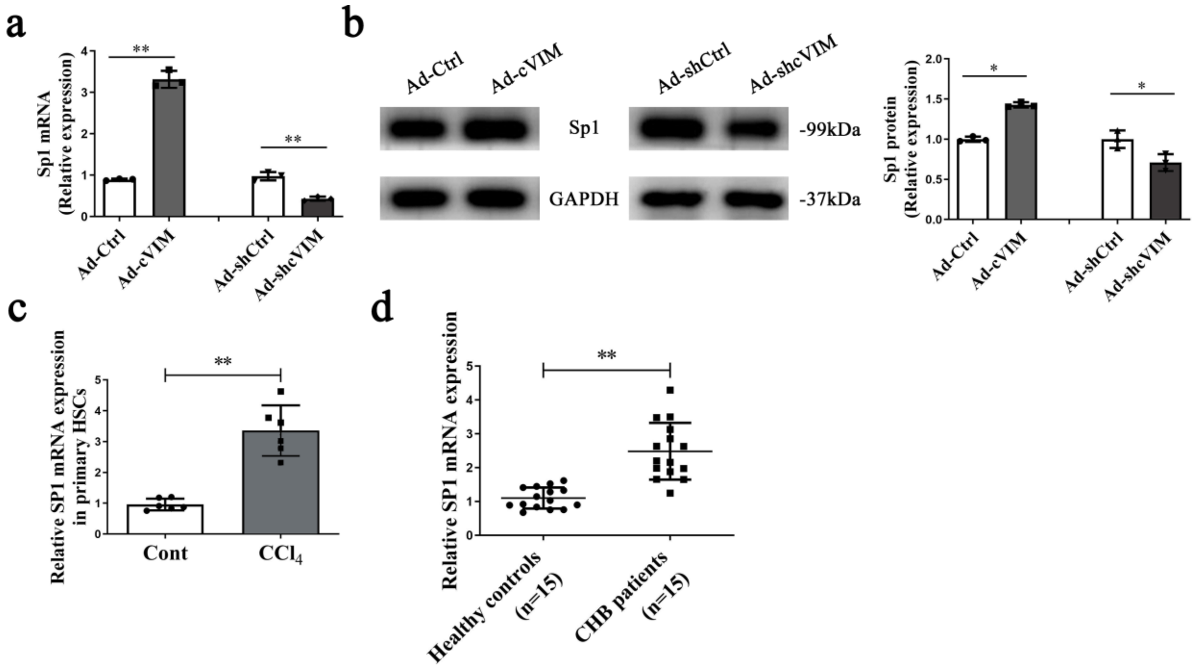

**Supplementary Fig.8 Effects of loss of cVIM in hepatocytes.** Primary hepatocytes were transduced with Ad-shcVIM for 48 h and then transfected with miR-122-5p/miR-9-5p inhibitor for additional 24 h. **a** mRNA expressions of TGFBR1 and TGFBR2 (n=3 per group). **b** Protein expressions of TGFBR1 and TGFBR2 (n=3 per group). **c** Cell proliferation (n=3 per group). **d** mRNA expression of Col1A1 (n=3 per group). **e** Protein expressions of p-smad2 and type I collagen (n=3 per group). **f** cVIM expression (n=3 per group). cVIM expression was examined in primary hepatocytes with Sp1 siRNA for 48 h. Each value is the mean ± SD of three independent experiments. *$P$<0.05, **$P$<0.01 and ***$P$<0.001.

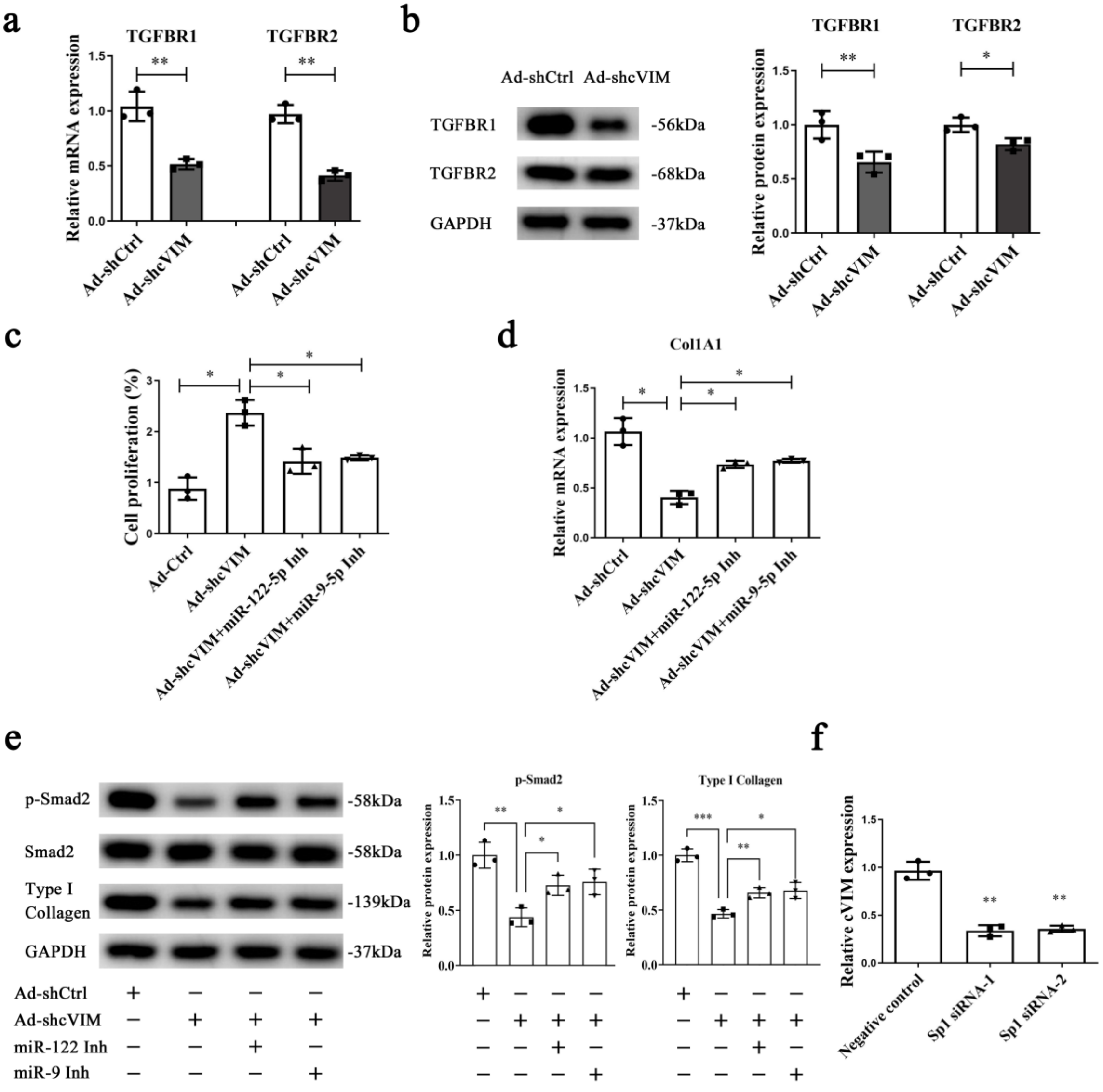

**Supplementary Fig.9 Expression of liver cVIM and serum cVIM in patients with liver fibrosis. (a and b)** All circRNA splices derived from human VIM gene were detected in the liver tissues from CHB patients (a, n=15 per group) and CHC patients (b, n=15 per group). **c** Serum cVIM levels in CHB patients (n=110 per group). **d** ROC curve analysis of serum cVIM for discriminating CHB patients with liver fibrosis (n=110 per group) from healthy controls (n=110 per group). **e** Serum cVIM levels in CHB patients with different fibrosis scores (n=110 per group). **f** The expression levels of miR-9-5p, miR-122-5p, TGFBR1 or TGFBR2 in liver samples from CHB patients (n=15 per group). Each value is the mean ± SD of three independent experiments. ***P*<0.01 compared with the control.

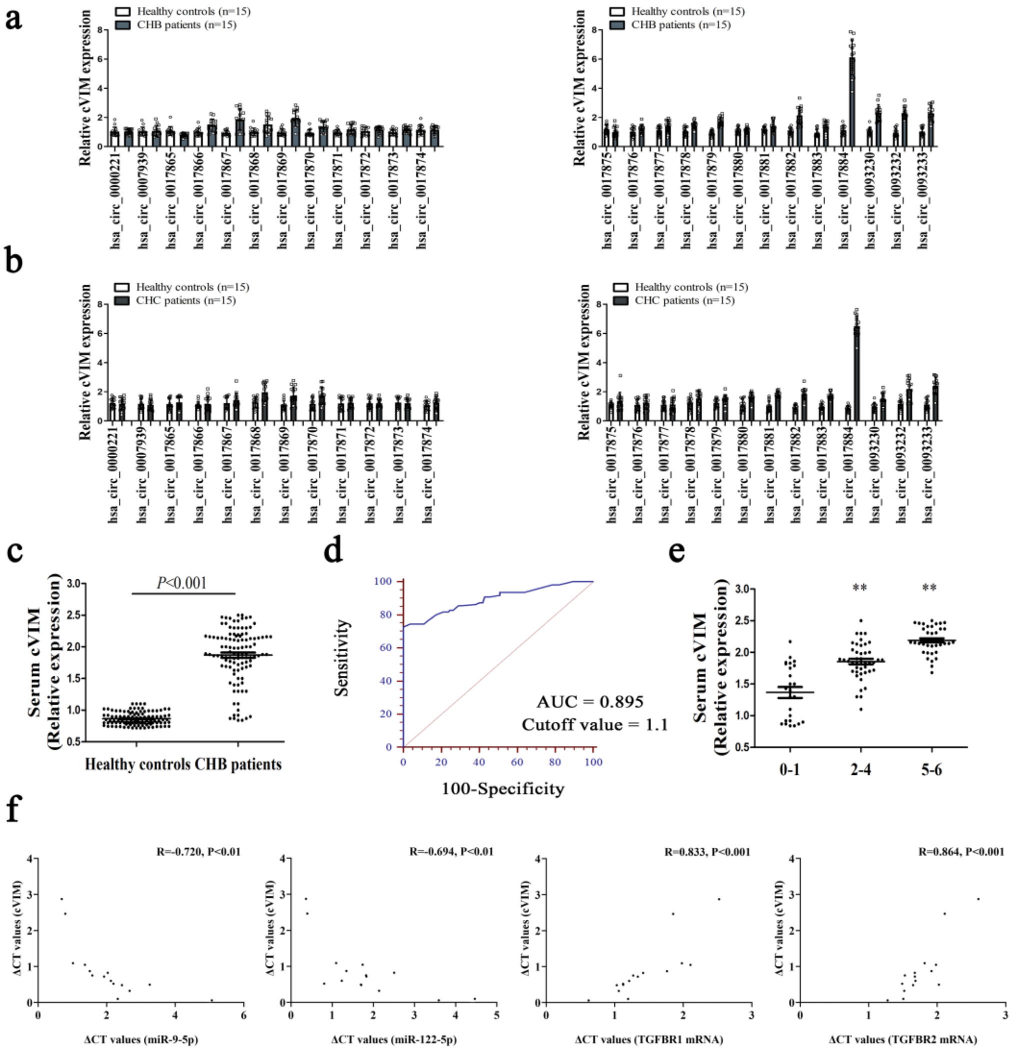

**Supplementary Fig.10 Expression of cVIM and mVIM after Sp1 overexpression.** Each value is the mean ± SD of three independent experiments. ***P*<0.01.

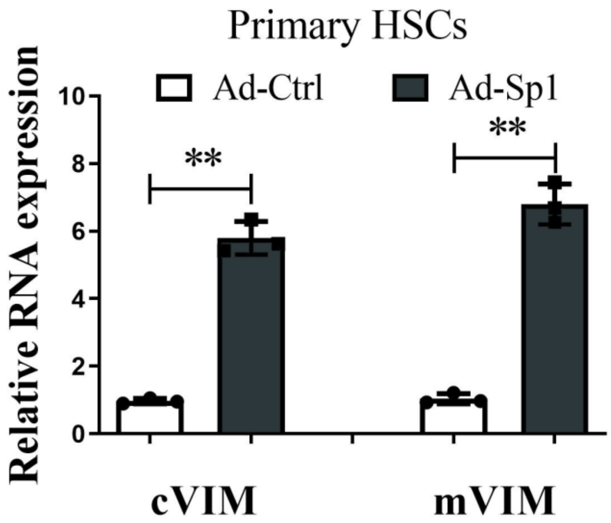

**Supplementary Fig.11 Roles of cVIM in hepatocytes. a** Trypan blue staining analysis (n=3 per group). **b** CCK-8 analysis (n=3 per group). **c** Cell cycle analysis (n=3 per group). Each value is the mean ± SD of three independent experiments. *$P$<0.05 and **$P$<0.01.

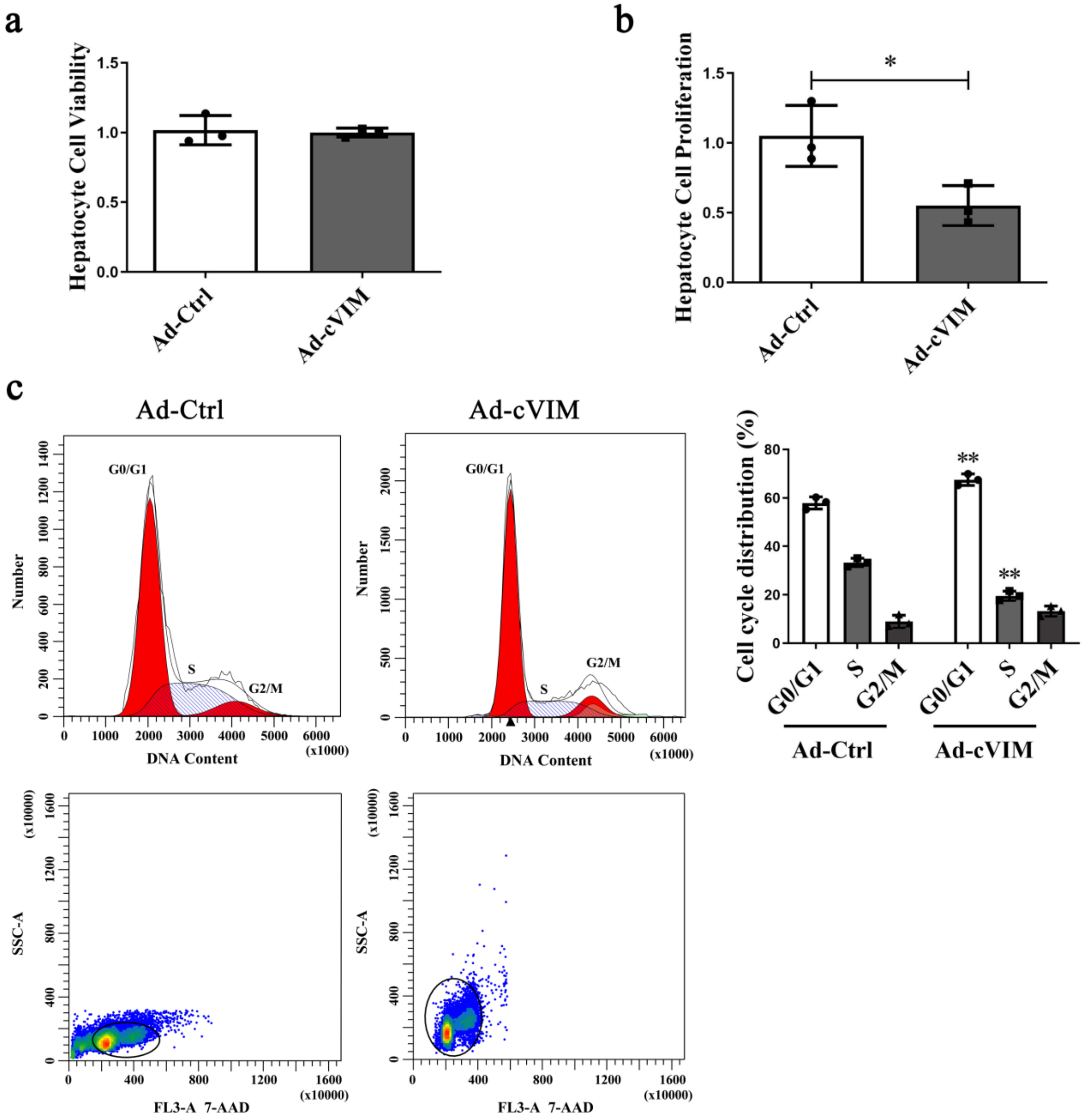

**Supplementary Fig.12 Unedited blot/gel images.**

Fig.3g

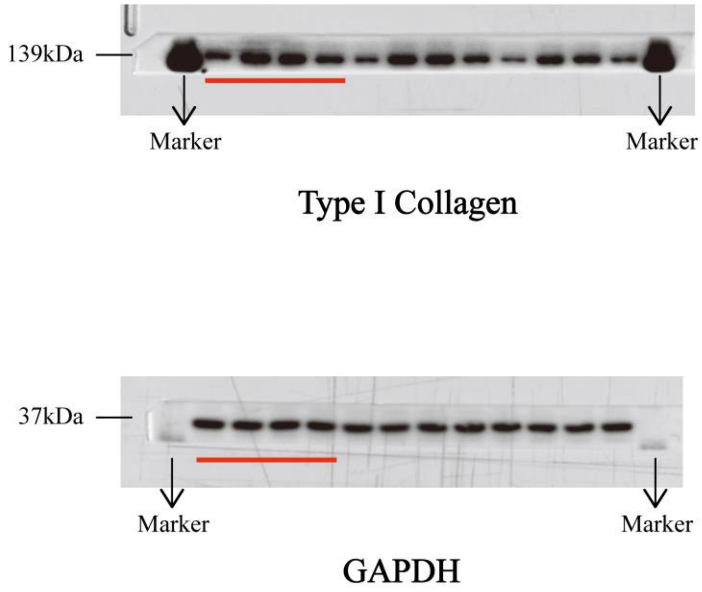

Fig.5b

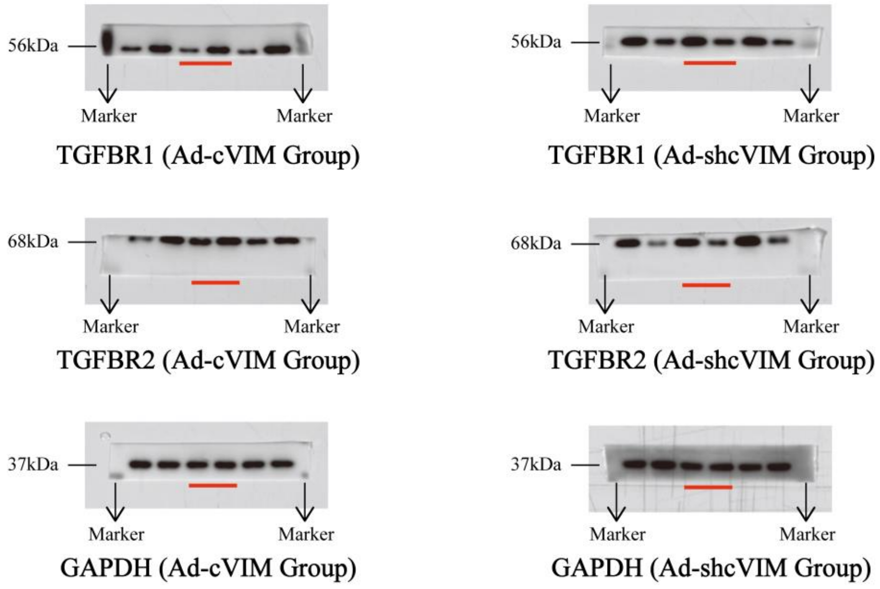


Fig.5c

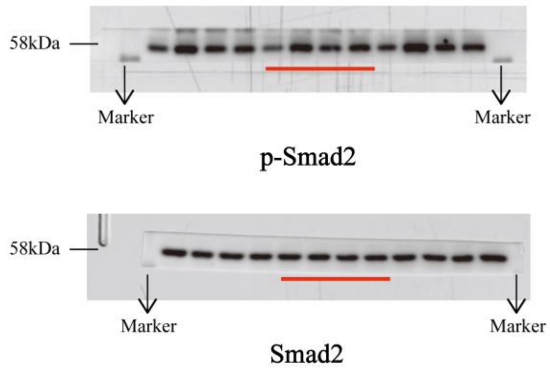


Fig.5f

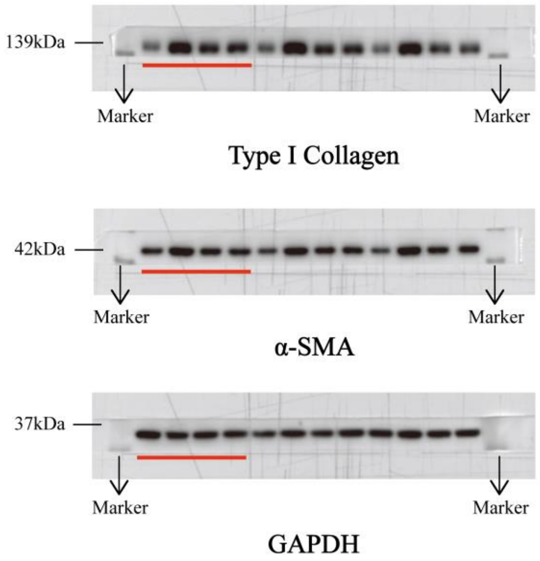


329

Fig.S3e

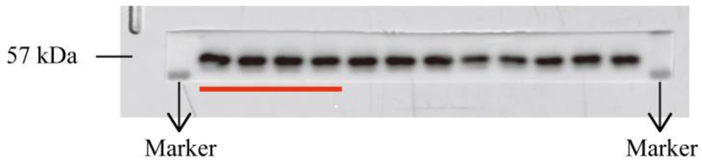


Vimentin

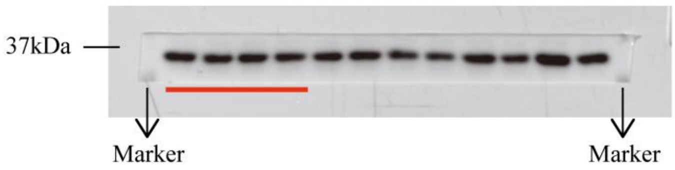


GAPDH

Fig.S6c

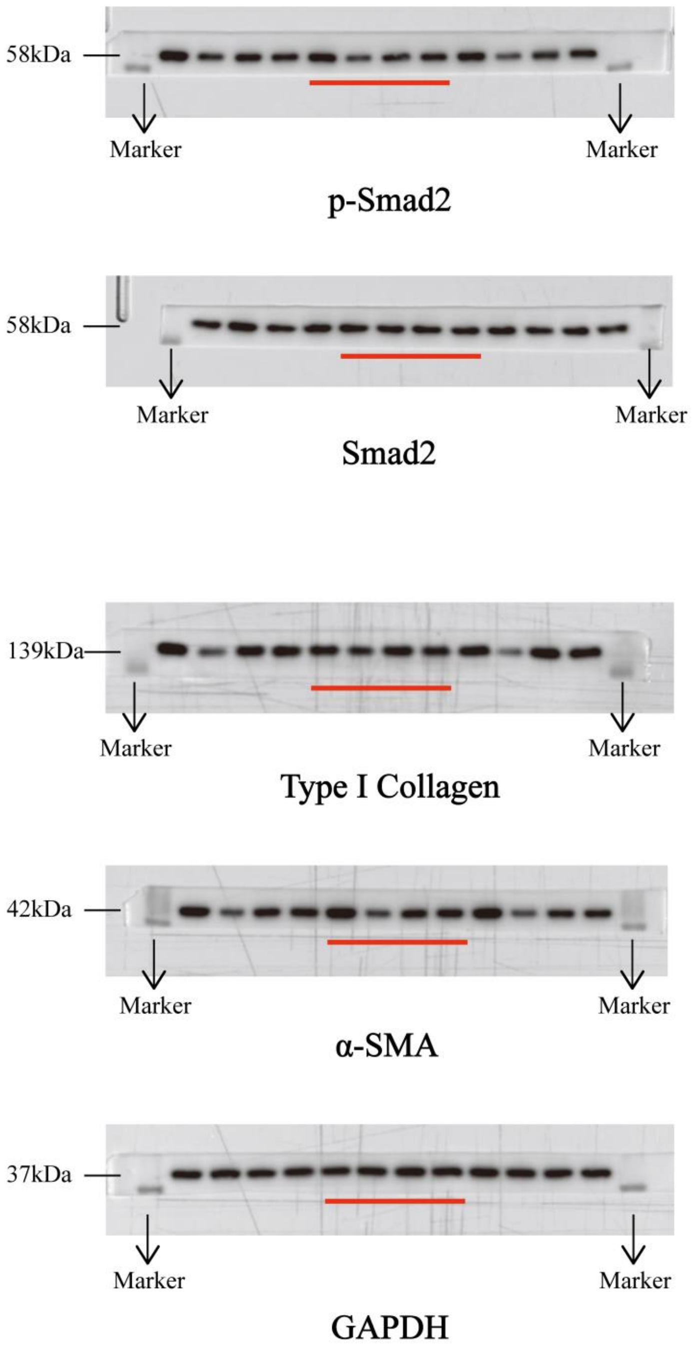
58kDa
Marker
Marker
p-Smad2
58kDa
Marker
Marker
Smad2
139kDa
Marker
Marker
Type I Collagen
42kDa
Marker
Marker
α-SMA
37kDa
Marker
Marker
GAPDH

Fig.S7b

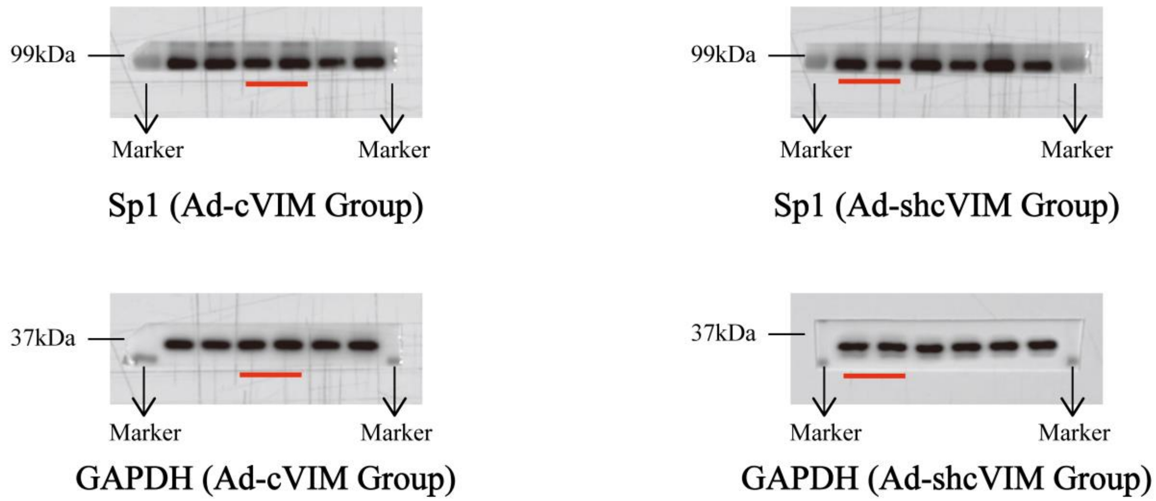
99kDa
Marker
Marker
Sp1 (Ad-cVIM Group)
99kDa
Marker
Marker
Sp1 (Ad-shcVIM Group)
37kDa
Marker
Marker
GAPDH (Ad-cVIM Group)
37kDa
Marker
Marker
GAPDH (Ad-shcVIM Group)

Fig.S8b

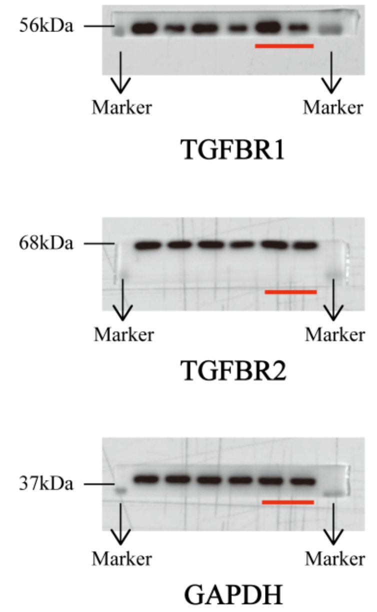


Fig.S8e

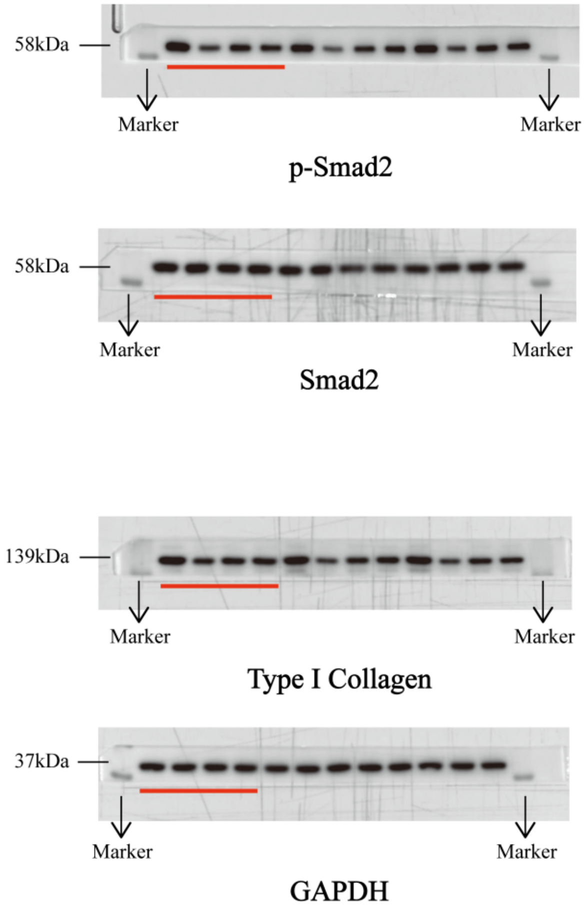

## Supplementary References


1 Yu, F. *et al.* Epigenetically-Regulated MicroRNA-9-5p Suppresses the Activation of Hepatic Stellate Cells via TGFBR1 and TGFBR2. *Cell Physiol Biochem* **43**, 2242-2252, doi:10.1159/000484303 (2017).

2 Miguel, V., Busnadiego, O., Fierro-Fernandez, M. & Lamas, S. Protective role for miR-9-5p in the fibrogenic transformation of human dermal fibroblasts. *Fibrogenesis & tissue repair* **9**, 7, doi:10.1186/s13069-016-0044-2 (2016).

3 Fierro-Fernandez, M. *et al.* miR-9-5p suppresses pro-fibrogenic transformation of fibroblasts and prevents organ fibrosis by targeting NOX4 and TGFBR2. *EMBO Rep* **16**, 1358-1377, doi:10.15252/embr.201540750 (2015).

4 Yu, F., Jiang, Z., Chen, B., Dong, P. & Zheng, J. NEAT1 accelerates the progression of liver fibrosis via regulation of microRNA-122 and Kruppel-like factor 6. *J Mol Med (Berl)* **95**, 1191-1202, doi:10.1007/s00109-017-1586-5 (2017).

5 Beaumont, J. *et al.* microRNA-122 down-regulation may play a role in severe myocardial fibrosis in human aortic stenosis through TGF-beta1 up-regulation. *Clin Sci (Lond)* **126**, 497-506, doi:10.1042/CS20130538 (2014).

6 Sun, Y. *et al.* miR-24 and miR-122 Negatively Regulate the Transforming Growth Factor-beta/Smad Signaling Pathway in Skeletal Muscle Fibrosis. *Mol Ther Nucleic Acids* **11**, 528-537, doi:10.1016/j.omtn.2018.04.005 (2018).

7 Zeng, C. *et al.* Identification of a novel TGF-beta-miR-122-fibronectin 1/serum response factor signaling cascade and its implication in hepatic fibrogenesis. *Oncotarget* **6**, 12224-12233, doi:10.18632/oncotarget.3652 (2015).
